# Single scan, subject-specific component extraction in dynamic functional connectivity using dictionary learning

**DOI:** 10.1162/IMAG.a.125

**Published:** 2025-09-02

**Authors:** Pratik Jain, Anil K. Sao, Bharat Biswal

**Affiliations:** Department of Biomedical Engineering, New Jersey Institute of Technology, Newark, NJ, United States; Rutgers School of Graduate Studies, Newark, NJ, United States; Department of Computer Science and Engineering, Indian Institute of Technology Bhilai, Bhilai, India

**Keywords:** individual differences, fMRI, dynamic functional connectivity, single scan, dictionary learning, brain fingerprint, common orthogonal basis extraction (COBE)

## Abstract

The study of individual differences in healthy controls can provide precise descriptions of individual brain activity. Following this direction, researchers have tried to identify a subject using their functional connectivity (FC) patterns computed by functional magnetic resonance imaging (fMRI) data of the brain. Currently, there is an emerging focus on investigating the identifiability over the temporal variability of the FC. Studies have shown that dynamic FC (dFC) can also be used to identify a subject. In this study, we propose a method using the dFC and a dictionary learning (DL) algorithm to extract the subject-specific component using a single fMRI scan. We show that once the dictionary is learned using a training set, it can be stored in memory and reused for other test subjects. Using Human connectome project (HCP) and Nathan Kline Institute (NKI) datasets, we showed that our proposed method can increase the subject identification accuracy significantly from 89.19% to 99.54% using the Schaefer atlas along with subcortical nodes from the HCP atlas. The effect of monozygotic and dizygotic twins on the subject identification was also analyzed, and the results showed no significant differences between the groups having twins and the group having unrelated subjects. This proposed method can aid in the extraction of the subject-specific components of dFC.

## 1 Introduction

Functional connectivity (FC) quantifies the temporal correlation between spatially remote neurophysiological events (Friston et al., 1993). FC can not only identify regions that are different between healthy controls and patient populations (Garrity et al., 2007; He et al., 2024; Xing et al., 2024) but also identify a target subject’s FC from a database of FCs (Finn et al., 2015). Identifying subjects from their FC boosted the concept of the “brain fingerprint” (Amico & Goñi, 2018; Van De Ville et al., 2021). Brain fingerprinting can help to identify the regions that are unique with respect to the individual, and by studying these regions, one can try to map the FC measures to individual behaviors (Finn et al., 2017). Secondly, analyzing the subject-specific FC can aid in mitigating the issues faced in differentiating clinical populations from healthy controls at the group level (Wolfers et al., 2018).

Researchers are now using signal processing algorithms to enhance the subject identifiability using FC. Amico and Goni demonstrated that individual identifiability in FC patterns can be enhanced by using approaches based on Principal component analysis (PCA) (Amico & Goñi, 2018). They proposed a new metric called differential identifiability ($I_{diff}$ ) that computes the difference between intra-subject similarity and inter-subject similarity. Using this metric, one could improve individual identifiability using PCA-reconstructed FC by showing increased $I_{diff}$ scores. Other studies (Cai et al., 2019; Kashyap et al., 2019; Qin et al., 2019) explored the use of dictionary learning (DL) algorithms to extract the individual identifiability in FC. These algorithms decompose the FC into a common and a subject-specific component such that the common component has information that is shared across the subjects, and the subject-specific component is unique to every subject (Jain et al., 2023). Cai et al. (2019) employed the K-singular value decomposition (K-SVD) algorithm to extract the subject-specific components from FC to enhance the prediction accuracy of behavioral scores. Moreover, a study by Qin et al. (2019) demonstrated that low-rank learning algorithms such as robust PCA (RPCA) enhanced individual differentiability in FC by using the FC and structural connectivity was computed using diffusion tensor imaging to predict behavior scores, including fluid intelligence and grip strength with higher accuracy.

Most studies have used the static FC, ignoring the temporal pattern of the FC to investigate the individual differences (Amico & Goñi, 2018; Chiêm et al., 2020; Finn et al., 2015; Kashyap et al., 2019; Qin et al., 2019). Sen and Parhi showed that dFC performs better as compared to the static FC for the prediction of behavioral scores such as fluid intelligence (Sen & Parhi, 2021). Moreover, Liu et al. (2017) observed that the strength, variability, and stability of the dFC showed divergent abilities in individual identification and cognitive prediction. Van De Ville et al. (2021) explored how the individual differences vary with time using dFC. One approach to computing the dFC is the sliding window approach. Instead of obtaining a single FC matrix for the entire duration of the scan, the sliding window computes the correlation of timepoints that lie in the window, which slides from the start to the end of the scan, thereby adding a temporal dimension to the scan. Van De Ville et al. (2021) demonstrated that there are certain time windows during which brains exhibit more unique patterns, which, if chosen properly, can contribute to better capturing the individual identifiability. Moreover, dFC has shown promise in capturing individual differences by predicting attention (Fong et al., 2019) and sensorimotor representations guiding body-environment interactions (Spadone et al., 2021). These studies highlight the potential of dFC to uncover subject-specific temporal patterns that may be related to cognitive processes, behavior, and individual traits.

**Fig. 1.**
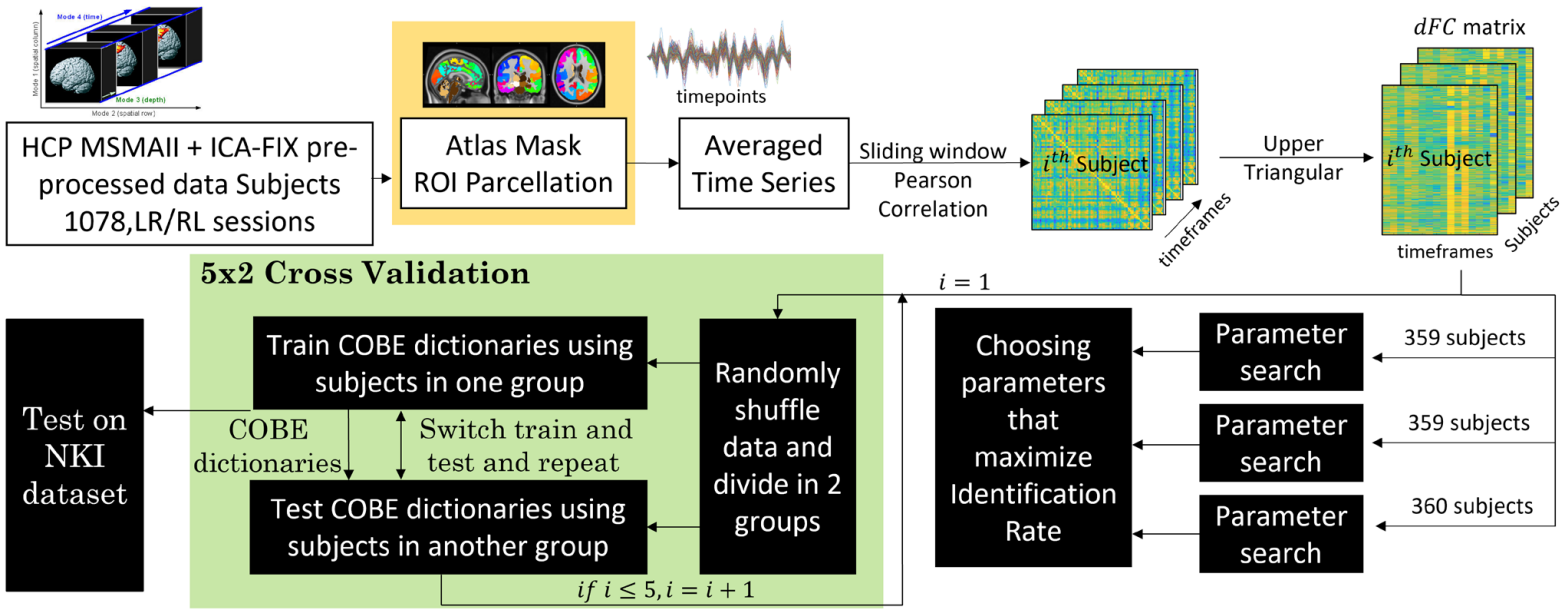
HCP MSMAII + ICA-FIX preprocessed data with Schaefer 400 atlas along with 19 sub-cortical regions, Seitzman 300 ROI, Power 264 ROI, Dosenbach 164 ROI, and AAL 116 ROI atlas is used to extract the region time series, and then the dynamic FC matrix is created. The complete dataset is divided into three groups (with 359, 359, and 360 subjects), and optimal dFC and COBE parameters are found by grid search and maximizing the Identification rate. Next, 5 x 2 cross-validation is used. Finally, the COBE dictionaries found during the cross-validation are tested on the dFC created using the NKI dataset.

In this study, we develop a method to extract the subject-specific dFC using the common orthogonal basis extraction (COBE) DL algorithm. The dFC is given as an input to the COBE algorithm, which gives us the common and subject-specific dFC. One of the key advantages of this method is its generalizability, that is, once the COBE dictionary is learned using at least a single scan of training subjects, it can be used to extract the subject-specific dFC components for other individuals who were not a part of the training set. This enables us to use our approach on new subjects without the need for retraining the COBE basis. To gain a comprehensive understanding of individual identifiability in dFC, the influence of seven resting state networks proposed by (Yeo et al., 2011) as well as the network formed by the regions that are not a part of the Yeo networks is investigated. This analysis provides insights into the contribution of different brain networks to the individual identifiability in dFC. We show that COBE is also robust across different datasets and MRI parameters. The COBE basis for the whole brain is then presented, which is used to get subject-specific components. We also tested the stability and generalizability of the algorithm. We used 1078 subject data from the Human connectome project (HCP) to demonstrate and validate our results and the Nathan Kline Institute (NKI) Rockland sample to test the COBE algorithm. Effect of monozygotic and dizygotic twins on the subject identifiability is also analyzed. The steps done in this study are summarized in Figure 1. Further to evaluate the proposed methods, the widely used dynamic differential identifiability $\left(dI_{diff}\right)$ proposed by Van De Ville et al. (2021) and the identification rate (IR) proposed by Finn et al. (2015) is used. By exploring the temporal dynamics of the subject-specific components using the COBE DL algorithm with dFC, our study aims to advance the field of individual identifiability and contribute to the development of personalized diagnostics.

## 2 Methods

### 2.1 Dataset and preprocessing

Publicly available datasets HCP and NKI Rockland Sample were used for the following study. All the data were collected by the respective sites after obtaining IRB from the participants.

#### 2.1.1 HCP dataset

The publicly available HCP dataset, consisting of 1078 subjects (583 F, mean age 28.86  ±  3.58 years), was used in this study. The resting-state images were acquired using a Siemens 3T Connectome Skyra scanner. Images were obtained with a TR of 720 ms with 2 mm isotropic voxels. We used the MSMAII + ICA-FIX preprocessed data for our analysis. The preprocessed HCP resting-state data underwent a comprehensive preprocessing pipeline (Glasser et al., 2013). The HCP preprocessing pipeline addresses several critical factors, including spatial distortions, subject motion correction through volume realignment, registration of fMRI data to structural information, bias field correction, global mean normalization, and brain masking, to isolate relevant data. Notably, the pipeline prioritizes minimizing smoothing effects from interpolation and explicitly avoids any deliberate volume smoothing, ensuring the preservation of fine-grained details in the fMRI data. All scans were normalized to the MNI 2 mm space, facilitating standardized analyses and comparisons across subjects. The data acquisition process involved two different encoding directions, left-right (LR) and right-left (RL). We considered both LR and RL encodings from the first run (rest1), thus obtaining two scans (also known as sessions) for every subject. The voxel time series were filtered using a bandpass Butterworth filter (using MATLAB commands “butter” and “filter”) of the 5th order in the range 0.01 to 0.10 Hz.

#### 2.1.2 NKI dataset

For exploring the generalizability with respect to different scanners, MRI parameter and preprocessing pipelines, we used resting-state data from 82 healthy subjects (55 F, mean age 41.83 ± 21.89) from the NKI dataset, that have two sessions acquired within duration of 1 year. Resting-state data were acquired from Siemens Magneton Trio Tim scanner with TR of 1.4 s and 2 mm isotropic voxels. Data were preprocessed using SPM12 in MATLAB. Realignment was performed, and subjects with excessive motion (max translation >3 mm and max rotation >3 degrees) were rejected. Thereafter, Skull was stripped from the anatomical scans and coregistered to the resting-state scans. The cerebrospinal fluid (CSF) and White Matter (WM) voxels were identified and the mean CSF, WM BOLD timeseries, was regressed out from every voxel in the brain along with the motion parameters. The data were then smoothed using 6 mm Gaussian kernel and finally, all the images were normalized to 2 mm MNI space. The voxel time series were filtered using a bandpass Butterworth filter (using MATLAB commands “butter” and “filter”) of the 5th order in the range 0.01 to 0.10 Hz.

### 2.2 Brain atlas

To parcellate the brain into distinct regions, a cortical parcellations atlas of 400 nodes created by Schaefer et al. (2018) was used along with 19 subcortical regions and three cerebellar regions provided by the HCP release (filename “*Atlas_ROI2.nii.gz*”). This combined atlas, referred to as the Schaefer-HCP, consists of 419 regions of interest (ROI). For each ROI, the time series corresponding to each voxel within the ROI was averaged, and an averaged time series was acquired for every region. This approach is commonly used to reduce the dimensionality of the data while preserving the essential information from each ROI. Based on previous findings (Jain et al., 2023), it was observed that the atlases with a larger number of ROIs and smaller voxel count per ROI tend to perform better in capturing individual identifiability when static FC is used. Thus, to further validate and compare the results for dFC across different brain atlases, the study also included analyses using the Seitzman 300 ROI atlas (Seitzman et al., 2020), Power atlas 264 ROI atlas (Power et al., 2011), and Dosenbach 160 ROI atlas (Dosenbach et al., 2010) combined with bilateral Amygdala and Para hippocampus (Di et al., 2013), which makes it a 164 ROI atlas. Supplementary Document includes results with the Automatic Anatomical Atlas (AAL). The inclusion of the AAL atlas provides insight into how the low ROI anatomical atlas affects subject identifiability. See Supplementary Figure S1 for a visual comparison of the different atlases used in this study.

### 2.3 Dynamic functional connectivity

Given the averaged time series for r ROIs, a sliding window method was used. Briefly, a window having a length of w was used to extract w timepoints from t to t+w where t starts from 1 and increases by a stride of S. Initially when t = 1 , the first w timepoints are extracted from the average time series of every region, and Pearson’s correlation is computed. This is the first timeframe of the dFC matrix. For the second timeframe t = S+1 and timepoints from S+1 to S+w are extracted, and Pearson’s correlation is computed. This is done till the last time point in the window is considered. This gives us a dFC matrix of size $\tilde{r}\times \tilde{w},$ where $\tilde{r}\;=\;r\times \frac{r-1}{2}$ is the number of elements in the upper triangular matrix of the r×r size Pearson correlation matrix, w is the window length, and $\tilde{w}\;=\;\left[\frac{T-w}{S}\right]+1$ is the number of time frames (T is the number of timepoints). This work also compares the results with 5 different window sizes (36, 72, 144, 288, 576 s). In addition, we also compared the different strides of 14.4, 36, 72 s.

Network-specific dFC is also computed by considering only the nodes that belong to a specific Yeo network (Yeo et al., 2011). The seven networks included Visual network (VN), Somato-motor network (SMN), Dorsal attention network (DAN), Ventral Attention network (VAN), Limbic network (LN), Fronto-Parietal network (FPN), and Default mode network (DMN). The sub-cortical ROIs were all grouped together and named the non-Yeo network (NYN). By choosing a subset of ROIs that only belong to a specific Yeo network, we have also reported the influence of the different networks on the $dI_{diff}$ and IR scores.

### 2.4 Common orthogonal basis transformation (COBE)

COBE is a DL algorithm first developed by Zhou et al. (2016). COBE works on multiblock data, where every subject is represented by a matrix instead of a vector. Given a group of matrices representing the different subjects, COBE attempts to find orthogonal basis that capture the common information across the different subjects. In this work, the COBE algorithm is given the dFC matrix corresponding to subjects in the training data.

During the training phase, the COBE algorithm takes as an input the dFC matrices of all training subjects ($dFC^{train})$ and the number of common components desired (*C*), and outputs the orthogonal common basis D of size $\tilde{r}\times C$ . During the testing phase, the subject-specific dFC ($dFC_{subject-specific}^{test}$ ) is extracted from the dFC of testing phase subjects ($dFC^{test}$ ) using the following equation:

$$dFC_{subject-specific}^{test}=dFC^{test}-dFC_{common}^{test}$$

$dFC_{common}^{test}$ is the common component of $dFC^{test}$ which is computed using the COBE dictionary D (learnt during training phase with training subjects) as follows:

$$dFC_{common}^{test}=D\times X^{test}$$

The coefficients of the COBE dictionary $X^{test}$ are found by least-squares estimates for an orthogonal dictionary and are represented as:

$$X^{test}=D^{T}\times dFC^{test}$$

### 2.5 Quantification of subject identifiability

#### 2.5.1 Dynamic identifiability ($dI_{diff}$ )

Quantifying individual differences relies on the assumption that the FC should overall be similar within scans of the same subject compared to scans between different subjects (Amico & Goñi, 2018). Based on this assumption, the differential identifiability ($I_{diff})$ metric was defined for static FC (Amico & Goñi, 2018). However, since for dFC, the FC varies with time, an extension of the differential identifiability termed as dynamic differential identifiability ($dI_{diff}$ ) was defined by Van De Ville et al. (2021). Data matrices ($dFC_{i}$) were created by concatenating the dFC of the $i^{th}$ session across all subjects, resulting in a 2-dimensional matrix of size $\tilde{r}\times (\tilde{w}.n_{sub})$ where $n_{sub}$ is the number of subjects. The correlation matrix formed by the correlation of two data matrices computed using different sessions is called the dynamic identifiability matrix (A = $corr(dFC_{1},dFC_{2})$ . This matrix can be seen as having subblocks of size $\tilde{w}\times \tilde{w}$ representing the correlation values between the different frames of subjects. The diagonal blocks represent the correlation values between the frames of the two different sessions of the same subject, and the non-diagonal blocks represent the correlations between the frames of two different sessions of different subjects. From the diagonal blocks, the frames are sorted based on the correlation values, which rank the frame based on the identifiability for a particular subject. The average correlation values of 1^st^ $j^{th}\;(j=1,2,3,\ldots ,\tilde{w})$ most identifiable frames corresponding to each diagonal blocks are averaged, and the resulting value is termed as $dI_{self}(j)$ , as these are the values of correlation between the dFC frames of the same subject. Similarly, values obtained by averaging the non-diagonal blocks are termed as $dI_{others}(j)$ , as they are correlations between dFC frames of different subjects. Finally, $dI_{diff}$ is obtained considering the first $j^{th}$ most identifiable frames as follows:

$$dI_{diff}(j)=dI_{self}(j)-dI_{others}(j)\;\;\;\;\;\;\;\;j=1,2,3,\ldots ,\tilde{w}$$

It is generally observed that $dI_{diff}$ is the highest when only 1 frame that is maximally correlated within the scans of the same subjects is used. One can view the $dI_{diff}$ score as a function of the number of frames that correspond to the maximum correlation values in the diagonal blocks. We reported results using just 1 frame that gives us the maximum value of $dI_{diff}$ and call it $dI_{diff}$ from here on out.

#### 2.5.2 Identification rate (IR)

The IR measures the accuracy with which the subject can be correctly identified from a different scan using correlations (Finn et al., 2015). The identity of a scan is assigned to the subject with which the scan is maximally correlated. Similar to the $dI_{diff}$ metric, the underlying assumption is that scans from the same subject are more similar compared to scans from different subjects. To compute the IR, the dynamic identifiability matrix (A) is utilized. The $i^{th}$ column of A represents the correlation of the $i^{th}$ dFC frame with the other dFC frames. Thus, the maximum correlation value across every column represents the dFC frame that is maximally correlated to the $i^{th}$ dFC frame. The subject whose frame gives the maximum correlation value is assigned to the $i^{th}$ frame. This predicted value is then compared to the original ground truth, as we know which frames belong to which subject, and a confusion matrix is created. Using this confusion matrix, the accuracy score is computed, which here is referred to as the IR. A higher IR indicates better individual identifiability, as it implies that the scans are more accurately associated with their corresponding subjects based on the similarity of their dFC patterns.

### 2.6 Test-train split

To robustly test the Subject-Specific components extracted from the COBE algorithm we followed a different train-test split for searching the parameters (the dFC window size and stride, and the number of common components (C) for COBE) and for cross-validation.

#### 2.6.1 Parameter search

The HCP dataset (1078 subjects) was divided into three blocks with 359, 359, and 360 subjects respectively in each block. For each block, the subjects were further divided into training and testing datasets such that the training dataset consisted of scans from a single session of 179 randomly selected subjects. This was done to address the variability from several sources. The training dataset was used to learn the COBE dictionaries. To ensure that the dictionaries learned could generalize to new subjects that were not a part of the training set, the test set consisted of data from subjects that were not used during training. We report the test IR scores observed for all three blocks. We then averaged the IR scores obtained from all the 3 blocks, and the parameters that achieved maximum IR scores were selected and used for the cross-validation analysis.

#### 2.6.2 5 x 2 cross-validation

The 5 x 2 cross-validation technique proposed by Dietterich (1998) was used to show that the COBE-derived subject-specific components are significantly better in subject identification. Briefly, the 1078 subjects were randomly divided into 2 halves, and one randomly chosen session (LR or RL) from the first half of subjects was used to train the dictionaries and two sessions of subjects from the second half were used to test the dictionaries. Next, one randomly chosen session (LR or RL) from the second half was used to train the dictionaries and two sessions of subjects from the first half were used to test the dictionaries. This was repeated five times, each time with a different group of subjects for the first and the second half. T values and corresponding p values were calculated as proposed by Dietterich (1998) demonstrating the difference between the $dI_{diff}$ and IR computed using the original dFC and the COBE derived subject-specific dFC. To correct for multiple comparisons for the 8 different resting state networks and the whole-brain network (a total of 9 multiple comparisons), we use Bonferroni correction. The Bonferroni-corrected alpha that is used is $\alpha_{corrected}=\frac{0.05}{9}=0.0056$ .

#### 2.6.3 Testing with NKI dataset

To explore how the COBE algorithm performs on new unseen datasets, we used the dictionaries learnt during the cross-validation phase. One set of dictionaries was obtained from every group; thus, we had 5×2=10 different dictionary sets. We used these 10 dictionary sets to obtain 10 different subject-specific dFC for the NKI dataset. To quantify the improvement due to COBE, the 5 x 2 test proposed by Dietterich (1998) was used to compare the $dI_{diff}$ and the IR scores extracted by the original dFC and the Subject-specific dFC extracted by COBE. To correct for multiple comparison for the 8 different resting-state networks and the whole-brain network, we use Bonferroni correction. The Bonferroni-corrected alpha that is used is $\alpha_{corrected}=\frac{0.05}{9}=0.0056$ .

### 2.7 Effect of twins and unrelated subjects

To investigate the effect of family relationships on subject identifiability, separate groups consisting of subjects having Monozygotic and Dizygotic twins were created. 147 pairs of Monozygotic twins and 74 pairs of Dizygotic twins were identified. Thus, we created a Monozygotic twin group with 294 subjects and a dizygotic twin’s group with 148 subjects. Another group of subjects that did not have any twins was created and named as the unrelated group (410 subjects). 5 x 2 cross-validation was used to find out the significant differences. It was ensured that for the monozygotic and dizygotic groups all the sub-groups created during the 5 x 2 cross-validation had both the twins.

## 3 Results

### 3.1 Parameter search

**Fig. 2.**
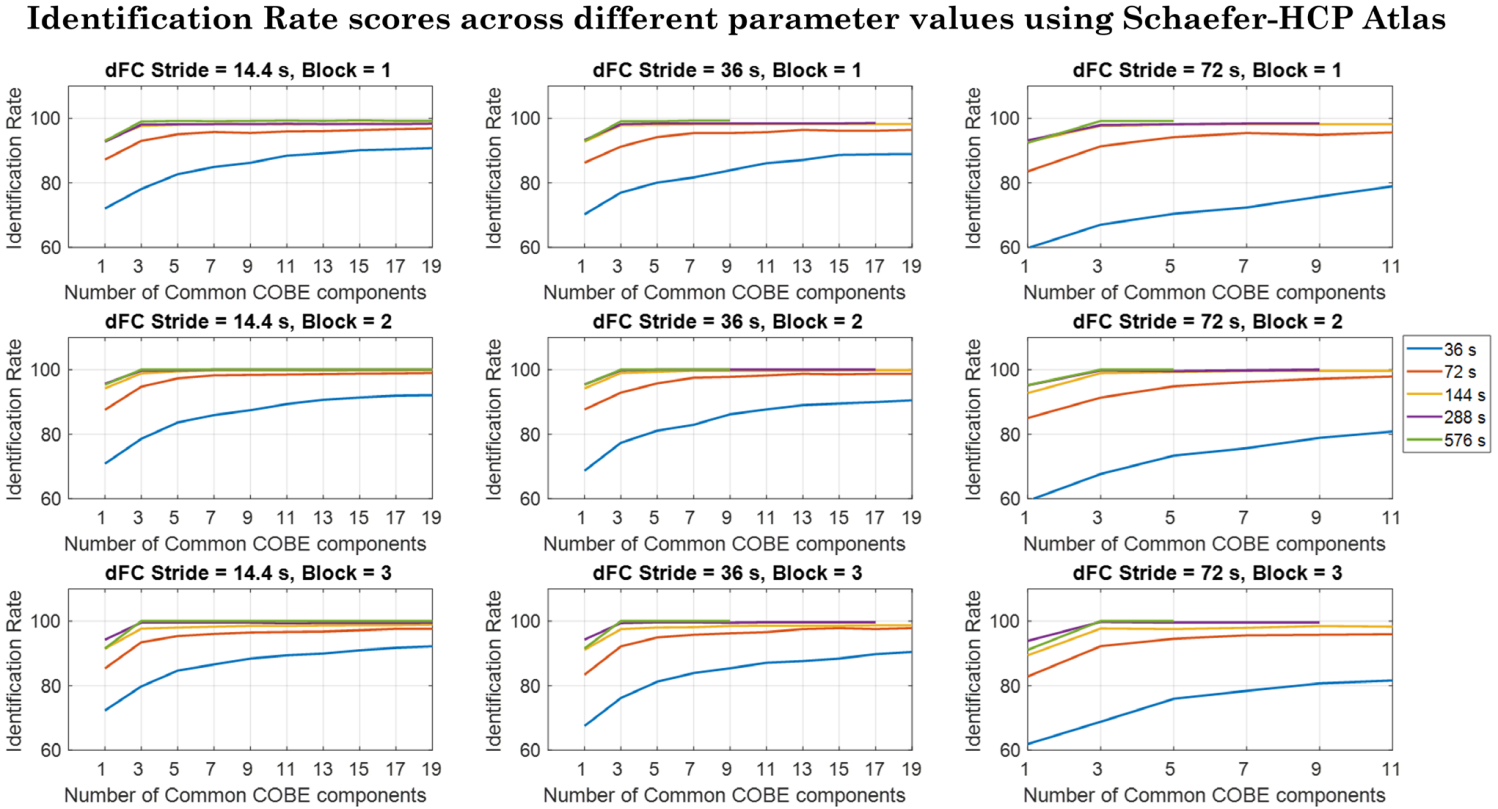
IR values computed for different values of window sizes, strides, and the number of common COBE components considering the whole-brain network when the Schaefer-HCP atlas was used. The parameters that maximized the IR scores were w=576s (800 timepoints), S=14.4s (20 timepoints), and C=15 .

One has to find the optimal window length and stride to compute the dFC. Parameter Search was done using the HCP dataset. In this study, we computed the dFC for window sizes of 36, 72, 144, 288, and 576 s and strides of 14.4, 36, and 72 s. All the dFCs corresponding to the training subjects were used to first train the COBE dictionaries independently for the 3 blocks, and then IR values were computed for the subjects not used while training. Figure 2 shows the IR values computed with the different values of the parameters described above for the whole brain for all the three blocks when the Schaefer-HCP atlas was used (Refer to the Supplementary Table 1 for the parameter search values for the network-specific dFC and the parameter check results performed for the Seitzman, Power, Dosenbach and AAL atlas). Figure 2 shows the IR values computed for the different blocks, different C values, different stride and different window sizes for the Schaefer-HCP using whole brain network (Refer to the Supplementary Figures S2-S26 for IR values corresponding to other atlases and networks). It was observed that the IR values increased by increasing the window size and saturated after window size of 144 s when whole-brain network was considered (see Fig. 2). Variation of the stride did not affect the results with window size greater than 144 s, but for the other window sizes a smaller stride value showed higher IR values. The COBE algorithm also has a parameter: the number of common COBE components (C). We varied C as from 1 to 19 in steps of 2. It was observed that the IR saturated when values of C > 3 were used for windows greater than equal to 144 s and C > 13 for window sizes less than equal to 72 s. C is also limited by the size of the dFC matrix. The maximum value that C can have is min(r,w). This is because we used canonical correlation to initialize the COBE basis. Canonical correlation uses the first C eigen vectors of the covariance matrix generated using the dFC matrices. Since the maximum eigen vectors possible are equal to $min(\tilde{r},\tilde{w})$ , we cannot have $C>\;min(\tilde{r},\tilde{w})$ . Thus, one can see the values on the x-axis are different across different strides. Also, the window sizes of 288 and 576 s stop at a lesser value of C than others. For instance, consider stride 36 s (S= 50 timepoints) and window size 576 s (w = 800 timepoints). $\tilde{w}=\left[\frac{1200-800}{50}\right]+1=9$ , and for the 419 ROI atlas, $\tilde{r}$ is fixed as 87571. Thus, for this configuration, C cannot be greater than min(87571,9)=9 . Therefore, there are no values for C greater than 9 when the stride is 36 s, and the window size is 576 s.

### 3.2 Significance of the subject specific dFC extracted by COBE

**Fig. 3.**
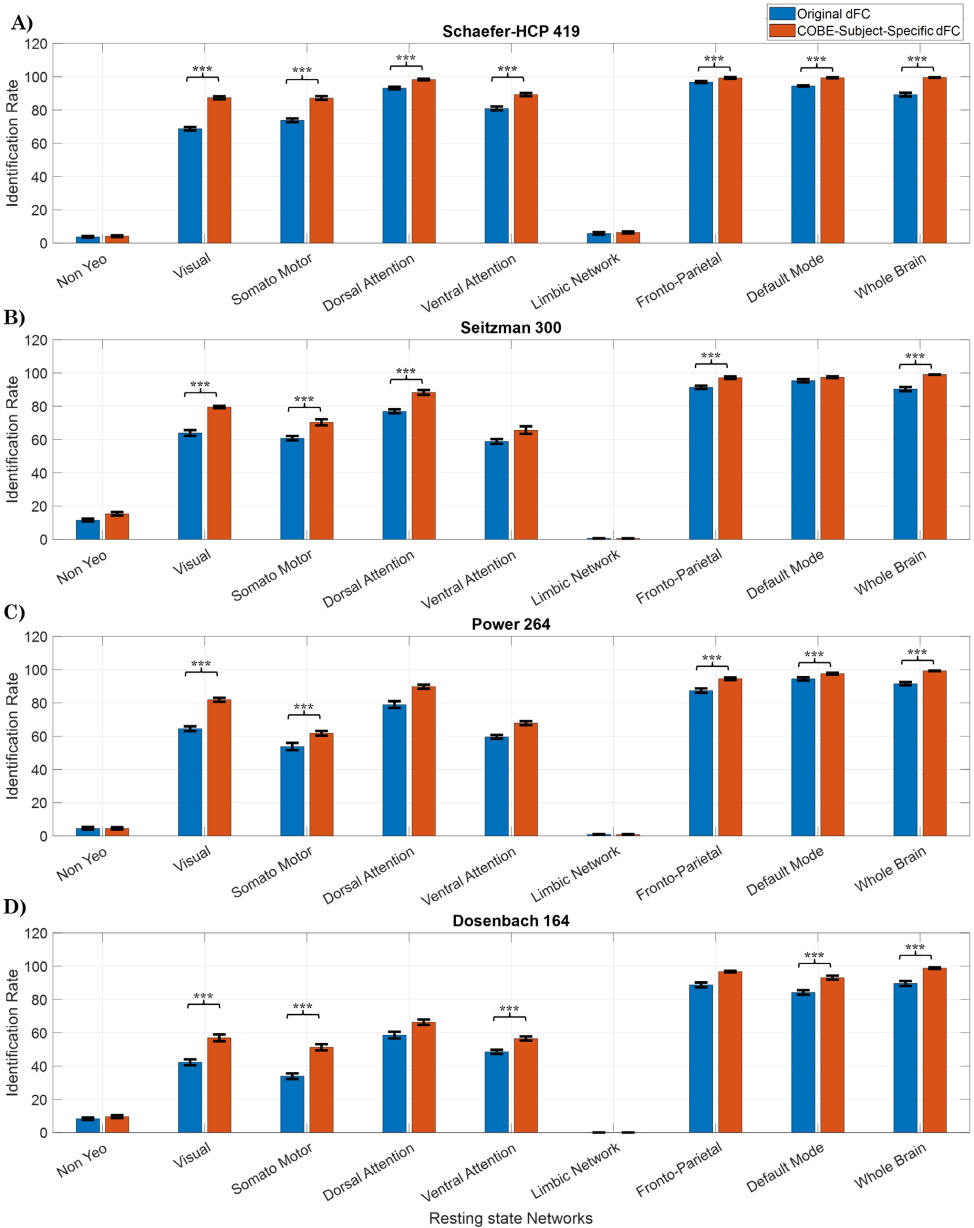
Cross-validation IR scores: 10 IR scores computed using the 5 x 2 cv method with (A) Schaefer-HCP, (B) Seitzman, (C) Power, and (D) Dosenbach atlases (Dosenbach atlas has no nodes in the limbic Network). The parameters that maximized the IR scores were chosen. T-values and corresponding p-values were computed using the 5 x 2 cv method. Error bars represent mean ± std across the 10 permutations. ***$p<\alpha_{corrected}$ ($\alpha_{corrected}=\frac{0.05}{9}=0.00556)$ .

**Fig. 4.**
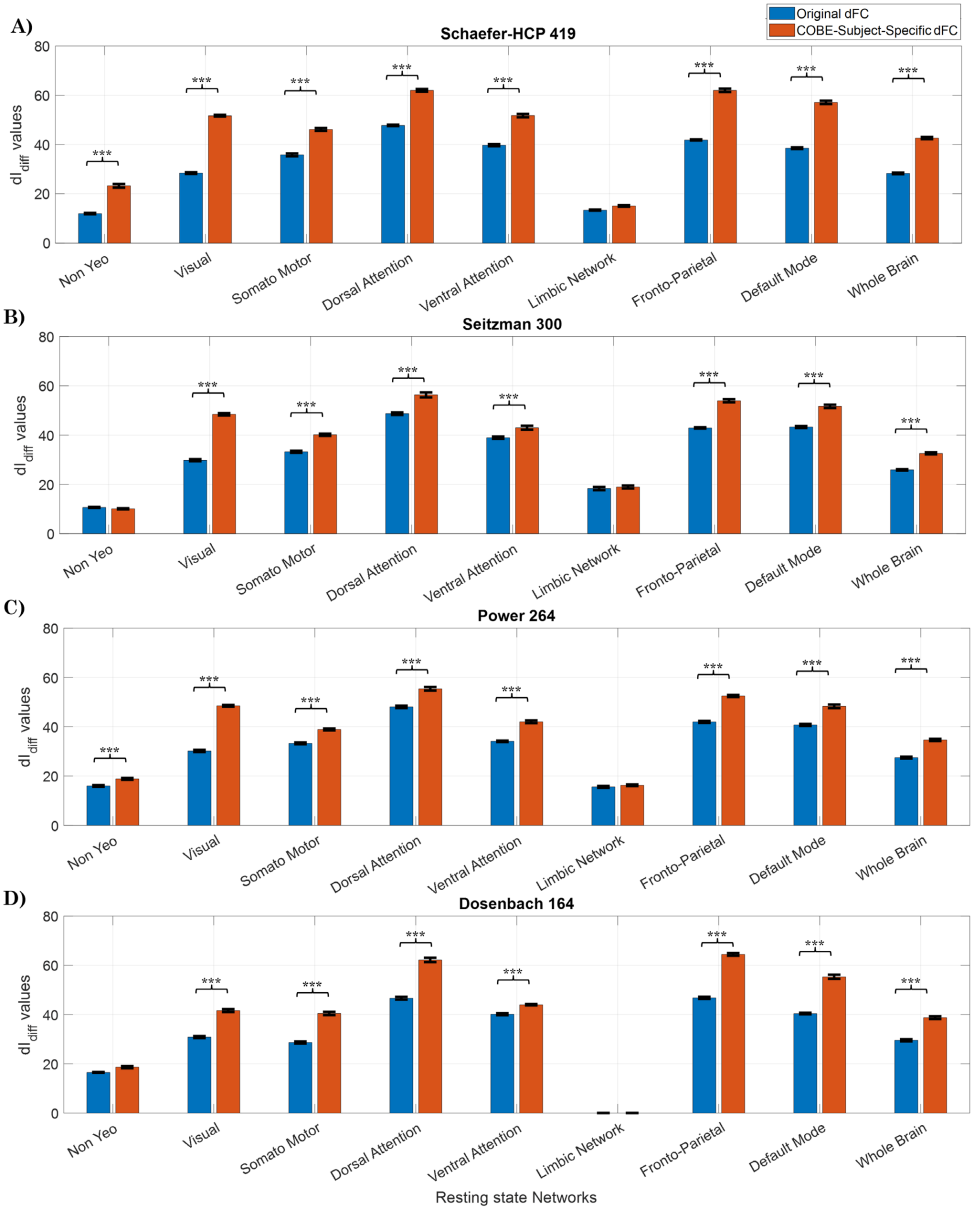
Cross-validation $dI_{diff}$ : 10 IR scores computed using the 5 x 2 cv method with (A) Schaefer-HCP, (B) Seitzman, (C) Power, and (D) Dosenbach atlases (Dosenbach atlas has no nodes in the limbic Network). The parameters that maximized the IR scores were chosen. T-values and corresponding p-values were computed using the 5 x 2 cv method. Error bars represent mean ± std across the 10 permutations. ***$p<\alpha_{corrected}$ ($\alpha_{corrected}=\frac{0.05}{9}=0.00556)$ .

Figure 3 shows the IR values across the different resting-state networks across the 5×2 permutations of cross-validation when the parameters that maximized the IR values were chosen. For instance, for the Schaefer-HCP atlas considering the whole-brain network, the parameters that maximized the IR score were 576 s window size (w=800 timepoints), stride of 14.4 s (S = 20 timepoints), and C = 15 (see Supplementary Table 1 for the optimal parameters used for the different resting-state networks across the different atlases). To remove any bias due to the training set, cross-validation was used. The mean IR over 5×2 trials for the dFC estimates and the subject-specific dFC extracted from COBE are shown in Figure 3. The error bars show the standard deviation of scores over 5×2 trials. Figure 3-(A, D) shows the IR when Schaefer-HCP, Seitzman, Power, and Dosenbach atlas are used, and Figure 4-(A, D) shows the $dI_{diff}$ scores when Schaefer-HCP, Seitzman, Power, and Dosenbach atlas are used (see Supplementary Figure S27 for IR values corresponding to AAL atlas).

From Figure 3-A, it can be observed that the mean IR scores obtained from the subject-specific dFC computed by the COBE algorithm are significantly higher (p < 0.0056) than the scores obtained from the original dFC across all the networks except NYN and LN. For instance, when the whole brain was considered, the mean ± std IR score for the original dFC was 89.19 ± 1.12%, and for the subject-specific dFC extracted using COBE it was 99.54 ± 0.13%. While comparing the subject-specific dFC among the resting-state networks, DMN achieved the highest IR value of 99.37 ± 0.29%, followed by the FPN (99.19 ± 0.54%) and DAN with IR of 98.31 ± 0.46%. The subcortical NYN (4.13 ± 0.51%) and the LN (6.41 ± 0.61%) achieved the lowest IR scores.

The trend with the $dI_{diff}$ score (see Fig. 4-A) is like that of the IR scores, with the Subject-Specific dFC achieving the highest score of 62.02 ± 0.54, 62.01 ± 0.67, and 57.12 ± 0.66 for the DAN, FPN, and DMN networks, respectively. On the other hand, NYN and LN achieved the lowest scores of 23.25 ± 0.73 and 15.07 ± 0.29, respectively.

On comparing the scores between the atlases (see Fig. 4), it can be observed that the $dI_{diff}$ values for the original dFC are comparable between the Schaefer-HCP, Seitzman, and Power atlases across the networks except NYN and VAN. For instance, for the FPN the $dI_{diff}$ scores were 42.93 ± 0.27 with the Seitzman atlas, 41.85 ± 0.25 with the Schaefer-HCP, and 41.98 ± 0.40 for Power atlas. For the DAN, the $dI_{diff}$ was 48.70 ± 0.49 for the Seitzman atlas, 47.77 ± 0.32 for the Schaefer-HCP, and 48.12 ± 0.48 for the power atlas. When all the nodes of both the atlas were used (whole brain), the scores were 25.90 ± 0.31 for the Seitzman atlas, 28.28 ± 0.30 for the Scheafer-HCP, and 27.46 ± 0.37 for the Power atlas. However, when the COBE algorithm is used to extract the Subject-Specific dFC, COBE is able to enhance the scores of Schaefer-HCP better than the Seitzman or the Power atlas. For example, the FPN $dI_{diff}$ scores for Subject-Specific dFC were 62.41 ± 0.67 for the Schaefer HCP atlas, but for the Seitzman atlas and Power atlas, it was only 53.95 ± 0.67 and 52.50 ± 0.44 respectively. Similarly with DAN, it was 62.02 ± 0.54 for Schaefer-HCP, 56.33 ± 1.02 for the Seitzman, and 55.41 ± 0.77 for the Power atlas. When the whole-brain network was considered, $dI_{diff}$ score was 32.58 ± 0.40 for Seitzman and 34.70 ± 0.45 for the Power whereas for Schaefer-HCP it was 42.64 ± 0.47. It is also interesting to note that the IR scores extracted from subject-specific dFC for the FPN, DMN, and the whole-brain network are very close to 100%, for all the three atlases, but only close to 100% with DAN for the Schaefer-HCP atlas.

The IR scores obtained using the Dosenbach atlas are less than those obtained using the other three atlases for all the networks except the FPN, DMN, and whole brain. Despite having 164 ROIs which is less than that of the other atlases, Dosenbach atlas is able to give high IR scores for FPN, DMN, and whole brain. Since for the Dosenbach atlas no ROIs were detected in LN, the scores corresponding to LN are zero. The COBE subject-specific dFC still has higher IR and $dI_{diff}$ scores as compared to the original dFC.

### 3.3 Effect of twins and unrelated subjects

**Fig. 5.**
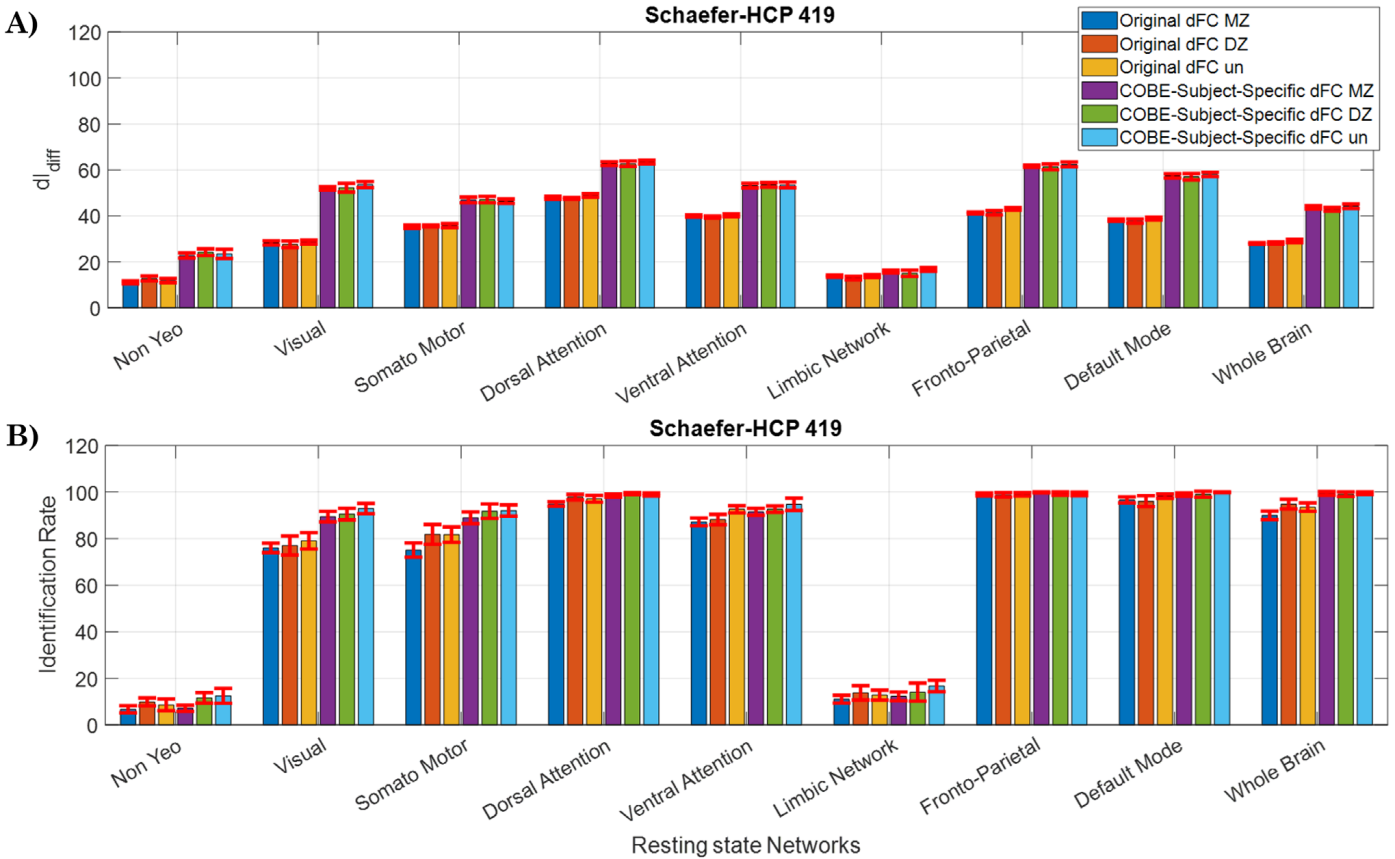
5 x 2 Cross-validation (A) $dI_{diff}$ and (B) IR scores for Monozygotic (MZ), Dizygotic (DZ), and unrelated subject groups (un).

Effects of twins and unrelated subjects were investigated by dividing the subjects into a monozygotic, dizygotic and unrelated subjects’ group. These groups were given as input to the COBE algorithm separately, and 5 x 2 cross-validation was performed. No significant differences were found in the $dI_{diff}$ or IR scores computed between the Monozygotic, Dizygotic, or unrelated subjects’ groups (See Fig. 5 for results obtained with the Schaefer-HCP atlas. Results for other atlases are in the Supplementary Figs. S29-S30). Although the COBE-derived dFC had higher scores as compared to the original dFC, there were no differences between the groups for the original dFC as well as the COBE-derived subject-specific dFC.

### 3.4 Testing with NKI dataset

**Fig. 6.**
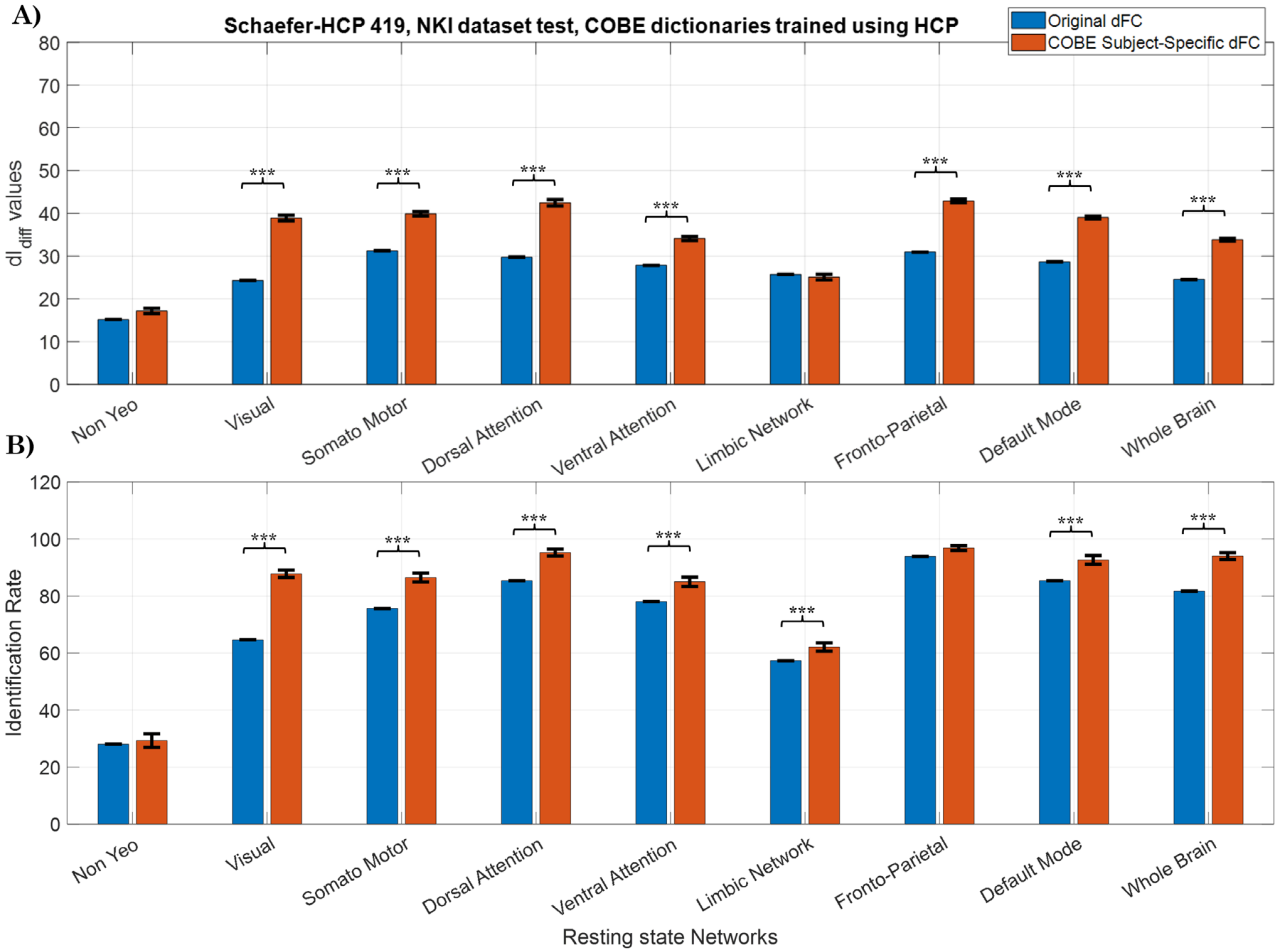
Testing (A) $dI_{diff}$ scores and (B) IR scores when the dFC created from NKI dataset was used. COBE dictionaries learnt using the HCP dataset were used to obtain the subject-specific dFC of the NKI dataset. T values and the corresponding p values were computed using the 5 x 2 cv method. Error bars represent mean ±  std of $dI_{diff}\;$ across the 10 COBE dictionaries set obtained from the HCP dataset. ***$p<\alpha_{corrected}$ ($\alpha_{corrected}=\frac{0.05}{9}=0.00556)$ .

The dictionaries learnt during the cross-validation phase with HCP dataset were used to obtain the subject-specific dFC from the NKI dataset. $dI_{diff}$ and IR scores obtained from the original dFC and the subject-specific dFC from the NKI dataset are shown in Figure 6. It was observed that the $dI_{diff}$ and IR scores obtained from subject-specific dFC computed from COBE were consistently higher than that of the original dFC except when the LN was considered. Notably, the original dFC results for the NKI dataset do not have error bars because the same subjects were used in all cases, with the only variation being the dictionary applied to extract subject-specific components. With the NKI dataset, the FPN network obtained the highest IR score of 94.91±0.8 followed by DAN with IR of 93.1±1.16 and DMN with IR of 91.95±1 . The IR obtained using the original dFC was 93.83 , 85.93 , and 87.44 for the FPN, DAN, and DMN networks respectively. For the $dI_{diff}$ scores, the subject-specific DAN network achieved the highest $dI_{diff}$ of 42.93±0.94 followed by FPN (40.99±0.52 ) and DMN (38.89±0.5 ). As observed with the HCP dataset, the NYN and the LN network obtained the lowest $dI_{diff}$ (18.65±0.76 , 24.71±0.99 ) and IR scores (30.14±1.74 , 60.26±1.25 ) respectively.

### 3.5 COBE basis

**Fig. 7.**
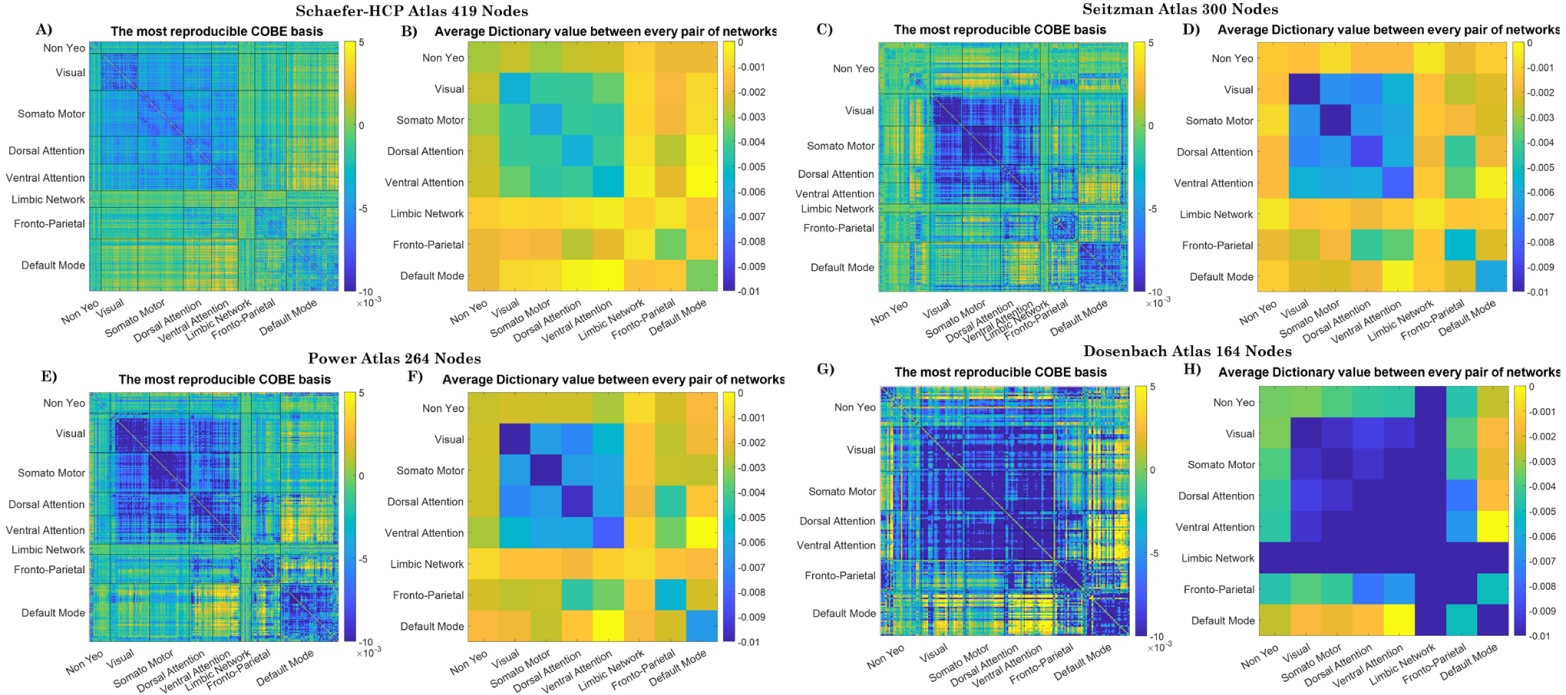
The most reproducible COBE dictionary basis and average dictionary value between every pair of the networks when (A-B) the Schaefer-HCP atlas, (C-D) when the Seitzman atlas (E-F), Power atlas, and (G-H) Dosenbach atlas was used.

The first dictionary basis was reproducibly found irrespective of the training data used to learn the COBE dictionaries across the 10 permutations. The COBE basis shows the weights that are applied on the dFC connections to get the common dFC component. Figure 7-A, C, E, and G shows the COBE basis computed using Schaefer-HCP, Seitzman, Power, and Dosenbach atlases respectively. To compare between the atlases, the ROIs are ordered based on the Yeo networks they correspond to. Since the size and location of the atlas nodes are different, the COBE basis looks quite different as well. However, there are similarities if the basis values are compared with respect to the Yeo Networks. The basis values within and between every pair of Yeo network were averaged, and the Average dictionary value between every pair of Yeo networks is demonstrated in Figure 7B, D, F, and H. Overall, it can be observed that the basis has negative values within and between the Somato-motor (SMN), ventral attention (VAN), DAN, and Visual network (VN) as compared to the other networks. Values close to 0 are seen in the interconnections between DMN/LN and the SMN, VAN, DAN, and VN. To quantify the similarity between the average Dictionary values across pairs of the five atlases, we correlated them and found an average significant correlation (p < 0.05) of 0.93 between dictionaries extracted using the five different atlases.

## 4 Discussion

This study proposes a method that extracts the subject-specific dFC and is able to enhance the subject identifiability by using a single scan for each subject with the help of the COBE algorithm. By training the COBE algorithm on the dFC computed using a single scan per subject, one can extract the subject-specific dFC of any new subject that was not used during the training of the COBE dictionary. Subject Identifiability, measured by $dI_{diff}$ and IR, was always higher when the COBE subject-specific dFC was used as compared to the original dFC. We compared the subject-identifiability across the resting-state networks and found the DAN, FPN, and DMN to obtain the highest and the NYN and LN to obtain the lowest $dI_{diff}$ and IR scores. Effect of twins and unrelated subjects was also explored. Comparisons were also made between the scores obtained between two different sets of parcellations, the Schaefer-HCP, Seitzman, and the AAL atlas. The results show that the subject identifiability computed using the $dI_{diff}$ scores by the original dFC using the 300 ROI Seitzman atlas is comparable to that of the 419 ROI Schaefer atlas; however, the COBE subject-specific dFC for the Schaefer Atlas is better as compared to that of the Seitzman atlas. The subject identifiability using atlas having lesser number of ROIs (Dosenbach) is the worse among the three atlases for networks other than DMN, FPN, and whole brain. No significant differences between the identifiability scores were found between the groups formed with the twins and the unrelated subjects group. The COBE dictionaries learnt using the HCP dataset was used to construct the subject-specific dFC for NKI dataset. It was observed that the subject-specific dFC extracted for NKI dataset had significantly improved individual differences, despite having different MRI parameters and preprocessing pipelines. Next, by analyzing the most reproducible COBE dictionary across the 10 cross-validation training and testing sets, we observed that the contribution of the intra-connections of the VN, SMN, DAN, and VAN to the common dFC component is larger than other connections as the basis values corresponding to these have greater magnitude as compared to other connections.

Using dFC, we are not only able to achieve enhanced subject identifiability but also study the identifiability with respect to time. We observed that the subject identifiability of the original dFC is poor when smaller windows are used to compute the dFC. The subject identifiability increases as the window size is increased and saturates at window size of 144 s, as also noted by Van De Ville et al. (2021). Across the different resting-state networks, we observed that DAN is the most identifiable, followed by FPN and DMN. For static FC, it was shown in the literature that the FPN and DMN are some of the best networks for subject identifiability (Amico & Goñi, 2018; Finn et al., 2015; Jain et al., 2023). We observed that it also holds true for the dFC. Moreover, the LN and the subcortical network, such as the NYN, seem to have the lowest subject identifiability.

In an earlier study, we have shown that different predefined brain atlases also affect individual differentiability scores (Jain et al., 2023). We observed that the FC computed using the brain atlases having a greater number of ROIs and a smaller number of voxels per ROI performed the best when static FC was used. Thus, we also performed the above analysis using the Seitzman 300 ROI, Power 264 ROI, and Dosenbach 164 ROI atlas, which has a fewer number of voxels per region as this is a spherical ROI-based atlas (the differences between the two atlases are mentioned in the Supplementary Fig. S1). Considering the original dFC, the mean value of $dI_{diff}$ scores across the 10 different permutations for training and testing subjects computed using the Seitzman atlas is similar to that of the Schaefer-HCP atlas used for all the networks except the NYN. Given that the Seitzman atlas has 300 nodes compared to the Schaefer HCP atlas, which has 419 nodes, the Seitzman atlas giving comparable $dI_{diff}$ and IR scores make it a better choice. However, when COBE is used to extract the Subject-Specific dFC, it is observed that the $dI_{diff}$ scores can be more enhanced in the case of Schaefer HCP atlas as compared to the Seitzman atlas. Thus, if the COBE algorithm is used to extract the subject-specific dFC, the Schaefer HCP is a better choice over the Seitzman atlas.

Atlases with less ROIs perform poorly for subject identification. Dosenbach atlas has less ROIs (164) but has smaller ROIs (spherical regions, see Supplementary Fig. S1). With the smaller regions, Dosenbach atlas outperformed the AAL atlas (116 ROIs) and gave results comparable to other atlases with a larger number of ROIs, especially for the DMN, FPN, and the whole-brain network. Results show that the subject identifiability using AAL atlas was worse compared to all the other atlases used in the study (see Supplementary Figs. S27 and S28). Interestingly, the AAL atlas achieved a $dI_{diff}$ score greater than that achieved with the other two atlases despite the IR scores being smaller. On further investigation, it was found that the non-diagonals of the identifiability matrix used to compute the $dI_{diff}$ scores had both positive and negative values making the average ($dI_{others}$ ) close to 0. Thus, the $dI_{diff}$ are misleading in this case and researchers should rely on the IR values as compared to the $dI_{diff}$ values.

We also investigated the effect of having monozygotic and dizygotic twins in the dataset. No significant differences were found on the $dI_{diff}$ or IR scores when data from only monozygotic twins or only dizygotic twins or unrelated individuals were used. Neither significant difference was found when the original dFC was used, not when the COBE-derived subject-specific dFC were used. This shows that COBE is unaffected by presence of subjects that are monozygotic or dizygotic twins, and it enhances the subject identifiability irrespective of them.

The generalizability of the COBE algorithm is further demonstrated by its application to a distinct dataset with differing MRI acquisition parameters and preprocessing pipelines. Specifically, the COBE dictionaries learned from the HCP dataset were successfully applied to enhance the dFC of the NKI dataset. This demonstrates the flexibility of the COBE framework in adapting to datasets with varying characteristics, making it a robust tool for improving subject-specific information. Moreover, this cross-dataset performance highlights the potential for COBE to bridge methodological and population differences between datasets, a critical step toward standardizing FC analyses across varying protocols. Future work could explore how dataset-specific factors, such as demographics or scanner variability, influence dictionary learning and the resulting subject-specific FC.

To further analyze the COBE basis concerning the different resting-state networks, we averaged the basis values obtained from the intra- and inter-connections within and between the resting-state networks (see Fig. 7-B for dictionaries computed using HCP data). The COBE basis shows how much weight is given to each of the connections between pairs of ROIs to make the common component of dFC across subjects. It was found that the magnitude of average values of the COBE basis within the SMN had the highest negative values, followed by VAN, DAN, and VN. This shows that there is more common information in these networks as compared to the other networks. The interconnections between the above-mentioned networks and the DMN had values close to 0. The positive and negative values in itself do not have a lot of significance, but they only tell that to create the common component, the magnitude of values within the negative connections needs to be subtracted from the magnitude of values of positive connections. Moreover, based on the atlas used to compute the dFC, the COBE basis values across the different networks also vary, but the trends remain to be similar as far as the Yeo networks are concerned.

While observing the dynamic identifiability matrix, it was found that COBE enhanced the subject identifiability by reducing the between-subject correlations. This is consistent with the idea of removing the components that are common between all the subjects. The reduction in off-diagonal correlations indicates that COBE effectively isolates subject-specific components, making each subject’s dynamic functional connectivity (dFC) more distinct from others. We also observed a moderate reduction in within-subject correlations. This can be explained by decomposing the original within-subject similarity into three sources: 1) True idiosyncratic signal that is unique to the individual, 2) Group‑level common signal that is shared across all subjects, and 3) Noise sources. When the common components are subtracted, the portion of the within-subject correlation they previously contributed disappears, so the correlation values in the diagonal decline (within-subject correlations). Crucially, the reduction is proportionally larger for non-diagonal correlation values (between-subject correlation) than for within-subject correlations; therefore, metrics such as $dI_{diff}$ and IR still rise, reflecting improved discriminability even though the absolute within-subject correlation is smaller. In other words, COBE intentionally trades a small loss of raw reproducibility for a larger gain in distinctiveness. Future work could combine COBE with denoising strategies that preserve the subject-specific signal while further attenuating noise.

The current work has some limitations. Firstly, COBE does not take into consideration the temporal contiguity; if the dFC frames are randomly scrambled and given to COBE, there would be no difference in the results as COBE considers the dFC frames independent. If an algorithm could also use temporal information, it could help enhance the individual differences. Furthermore, our study was limited to resting-state data only. Researchers have also analyzed the individual differences with naturalistic stimuli like movie watching (Di et al., 2023; Mittal et al., 2024; Vanderwal et al., 2017). Future studies should analyze the movie-watching dataset using DL algorithms and observe if the individual differences in the networks vary with respect to the movie shown during the scan.

## 5 Conclusion

In this study, we extract the subject-specific components from dFC corresponding to a single resting-state fMRI scan for each subject using the COBE algorithm. The COBE dictionary, once learned by the subjects used in the training dataset, can be stored and used to extract the common and subject-specific dFC of any new test subject. It was also found that the Schaefer-HCP atlas, consisting of 419 nodes, is a better choice of atlas as compared to the Seitzman, Power or Dosenbach atlases if the COBE algorithm is used to extract subject-specific components. However, if the FPN, DMN, or whole-brain network is used, then all the functional atlases used in the study perform similarly. Regardless of the atlas used, the FPN, DMN, and whole-brain networks gave the highest IR scores. For Schaefer-HCP and Seitzman atlas, the IR scores for the above-mentioned networks were very close to 100% with much less variability across the 5 x 2 cross-validation sets used to train the COBE dictionary, which means almost every subject was correctly identified when these networks were used. Moreover, no significant effect of twins was found on subject identifiability. Overall, this study can transition the field of fMRI from group-level analysis to individual or subject-specific analysis by using methods like COBE that can enhance the identifiability of subjects.

## Supplementary Material

Supplementary Material

## Data Availability

The HCP datasets are open-access data that are available for other researchers to download using connectome DB software from www.humanconnectome.org. This study along with the IRB was obtained by Washington University at Saint Louis. The NKI dataset is also freely available by using the bash or python script available at http://rocklandsample.org/accessing-the-neuroimaging-data-releases. Similarly, the data and the IRB for this study were obtained by Nathan Kline Institute at Orangeburg, NY. All the codes used in this work can be accessed from the GitHub repository https://github.com/Brain-Connectivity-Lab/Single-Scan-Subject-Specific-component-extraction-in-dFC-using-DL
