# Supplementary material for: Single scan, subject-specific component extraction in dynamic functional connectivity using dictionary learning
Source: Imaging Neurosci (Camb). 2025 Sep 2;3:IMAG.a.125. doi: 10.1162/IMAG.a.125 (PMC12406055; doi:10.1162/IMAG.a.125)
Supplement: Supplementary Material [file IMAG.a.125_supp.pdf]

Single Scan, Subject-Specific component extraction in dynamic functional connectivity using Dictionary Learning.

Pratik Jain[1,2], Anil K. Sao[3], Bharat Biswal[1]
[1]New Jersey Institute of Technology, Newark, NJ, US, [2]Rutgers School of Graduate Studies, Newark, NJ, US, [3]Indian Institute of Technology Bhilai, Bhilai, India.

Brain Atlas

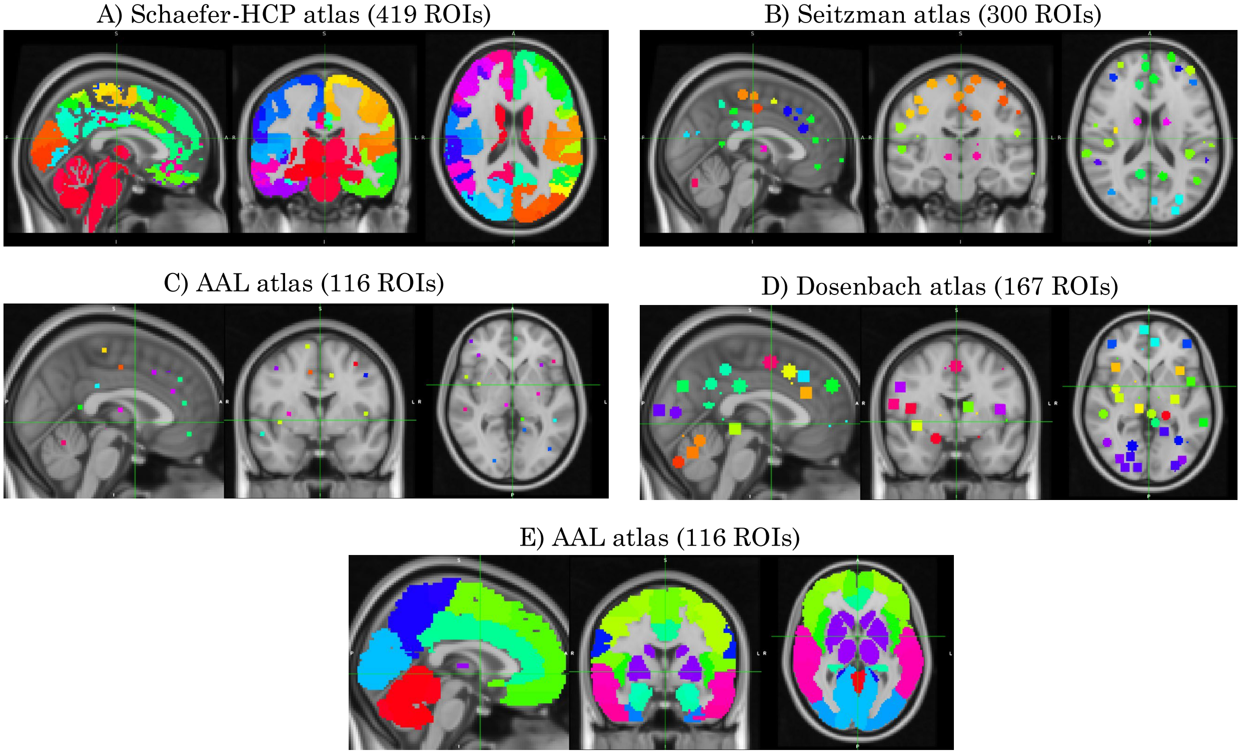

Figure S1: Brain Atlases A) Schaefer HCP atlas covering almost all voxels in the gray matter, B) Seitzman atlas, C) Power atlas, D) Dosenbach atlas with spherical ROIs C) AAL atlas covering almost all voxels in gray matter but having less number of ROIs.

Figure S1 shows the brain atlases Schaefer HCP, Seitzman, Power and Dosenbach and AAL. The Schaefer HCP atlas covers almost all the voxels in gray matter; thus, it has bigger regions of interest (ROI) however, the Seitzman, Power and Dosenbach atlas has spherical ROIs. Here one can see that the ROIs are small and not all voxels in the gray matter are included in the atlas. AAL atlas covers all the voxels but just has 116 ROIs, so the ROIs in AAL are far bigger than those in Schaefer-HCP.

Parameter Search values for the network specific dFC are shown in Figure S2-S9

Table 1: Optimal Parameters that maximize the IR

Atlas	Networks	Window (s)	Stride(s)	COBE (C)

Schaefer-HCP	Whole brain	576	14.4	15
	Non-Yeo	576	14.4	11
	Visual	576	14.4	19
	Somato-Motor	576	14.4	17
	Dorsal Attention	576	14.4	19
	Ventral Attention	576	14.4	19
	Limbic network	576	14.4	17
	Fronto-Parietal	576	14.4	15
	Default-Mode	576	14.4	17
Seitzman	Whole brain	576	36	3
	Non-Yeo	576	14.4	15
	Visual	576	14.4	19
	Somato-Motor	576	14.4	19
	Dorsal Attention	576	14.4	19
	Ventral Attention	576	14.4	19
	Limbic network	576	14.4	1
	Fronto-Parietal	576	14.4	19
	Default-Mode	576	36	7
Power	Whole brain	576	36	9
	Non-Yeo	576	36	1
	Visual	576	14.4	17
	Somato-Motor	576	14.4	19
	Dorsal Attention	576	14.4	19
	Ventral Attention	576	14.4	19
	Limbic network	576	72	1
	Fronto-Parietal	576	14.4	19
	Default-Mode	576	14.4	19
Dosenbach	Whole brain	576	14.4	17
	Non-Yeo	576	14.4	19
	Visual	576	14.4	19
	Somato-Motor	576	14.4	19
	Dorsal Attention	576	14.4	15
	Ventral Attention	576	14.4	19
	Limbic network	-	-	-
	Fronto-Parietal	576	14.4	19
	Default-Mode	576	14.4	19
AAL	Whole brain	576	14.4	19
	Non-Yeo	576	14.4	19
	Visual	576	14.4	19
	Somato-Motor	576	14.4	11
	Dorsal Attention	-	-	-
	Ventral Attention	576	14.4	1
	Limbic network	576	36	9
	Fronto-Parietal	576	36	7
	Default-Mode	576	14.4	17

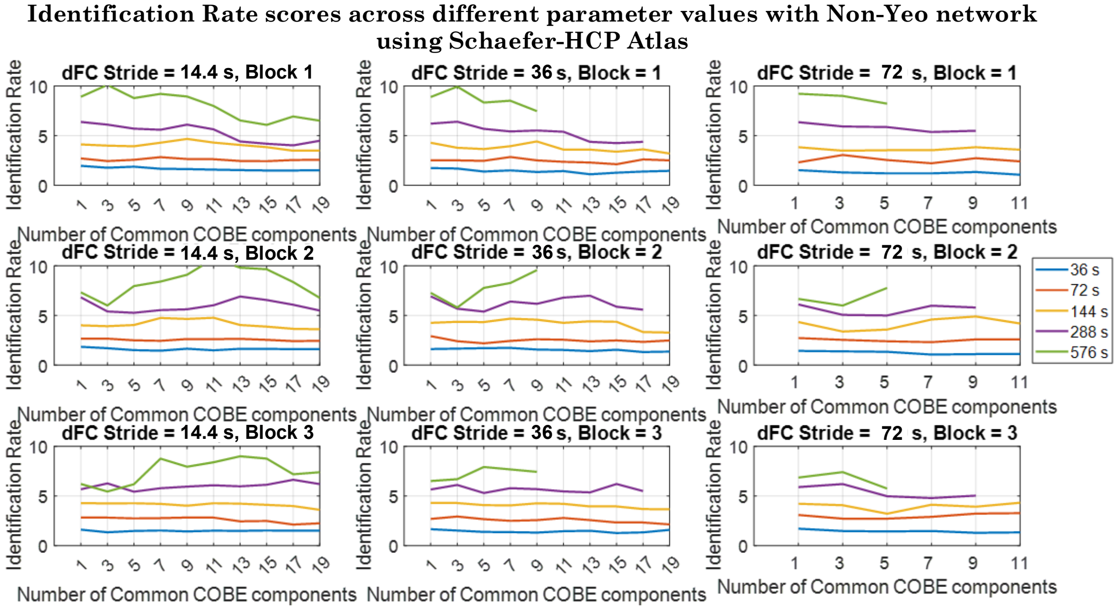

Figure S2: IR values computed for different values of window sizes, strides, and the number of common COBE components considering the Non-Yeo network using Schaefer-HCP atlas.

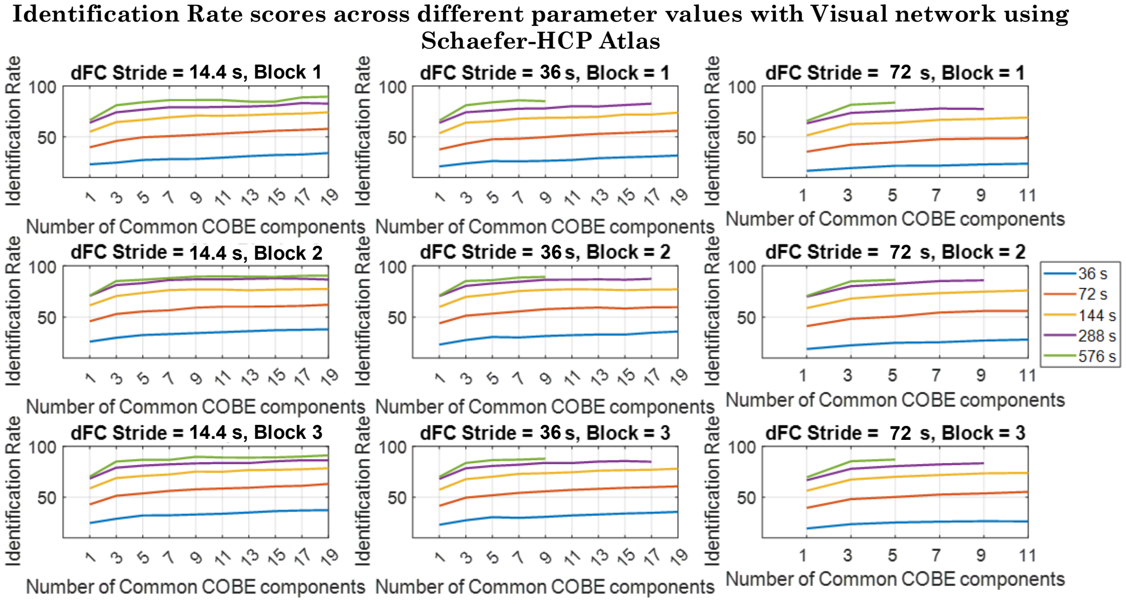

Figure S3: IR values computed for different values of window sizes, strides, and the number of common COBE components considering the Visual network using Schaefer-HCP atlas.

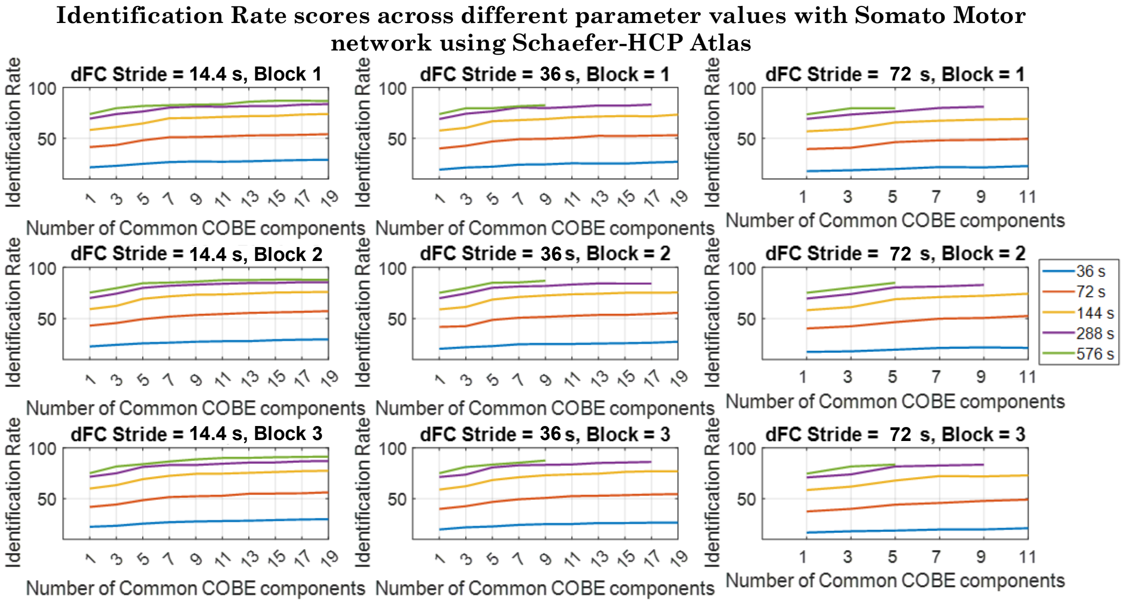

Figure S4: IR values computed for different values of window sizes, strides, and the number of common COBE components considering the Somato-Motor network using Schaefer-HCP atlas.

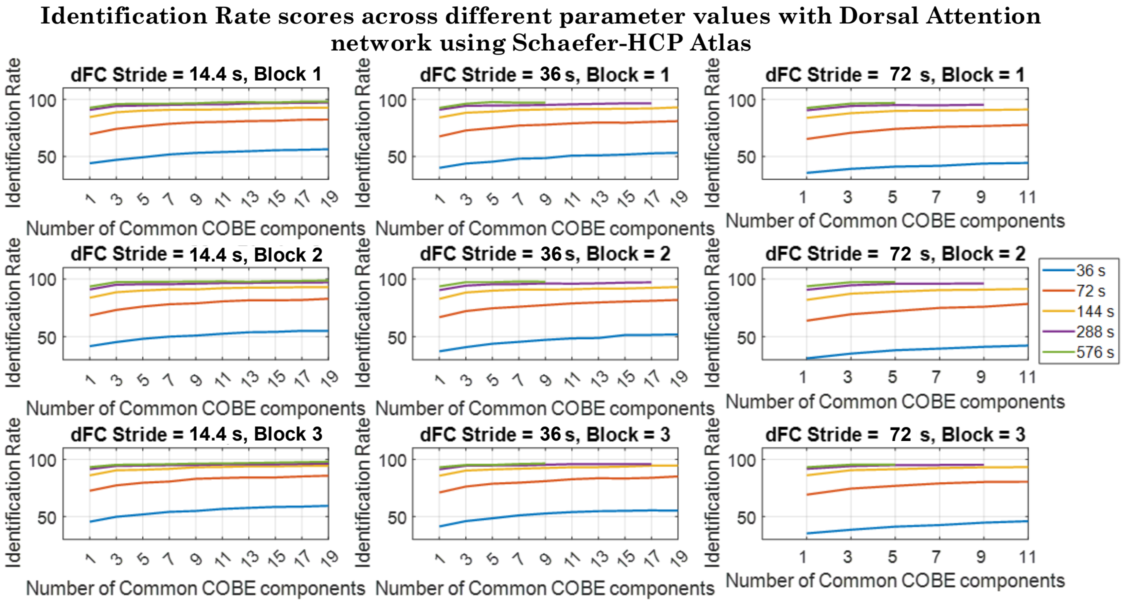

Figure S5: IR values computed for different values of window sizes, strides, and the number of common COBE components considering the Dorsal Attention network using Schaefer-HCP atlas.

Identification Rate scores across different parameter values with Ventral Attention network using Schaefer-HCP Atlas

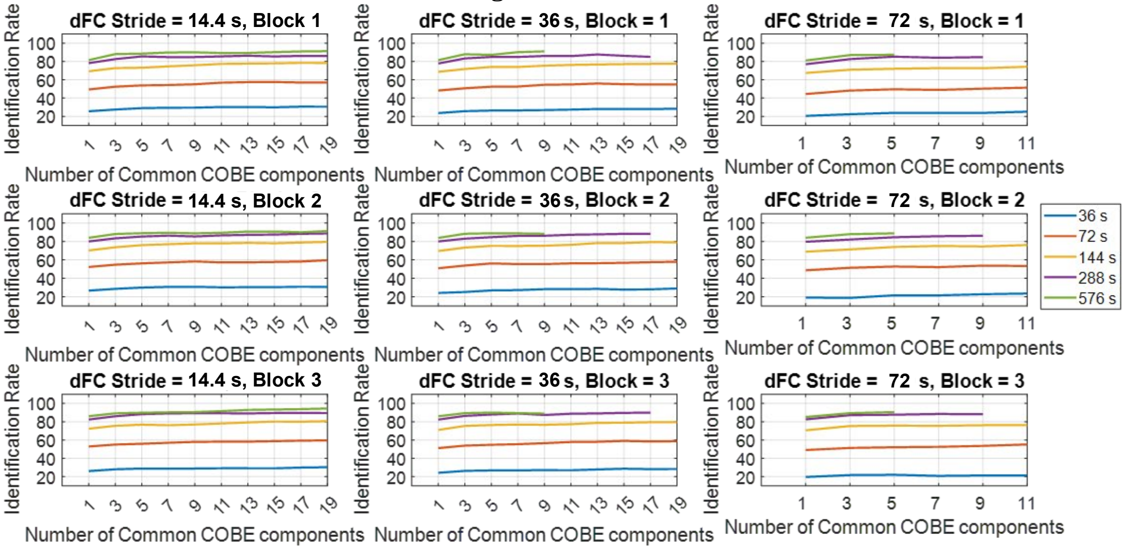

Figure S6: IR values computed for different values of window sizes, strides, and the number of common COBE components considering the Ventral Attention network using Schaefer-HCP atlas.

Identification Rate scores across different parameter values with Limbic network using Schaefer-HCP Atlas

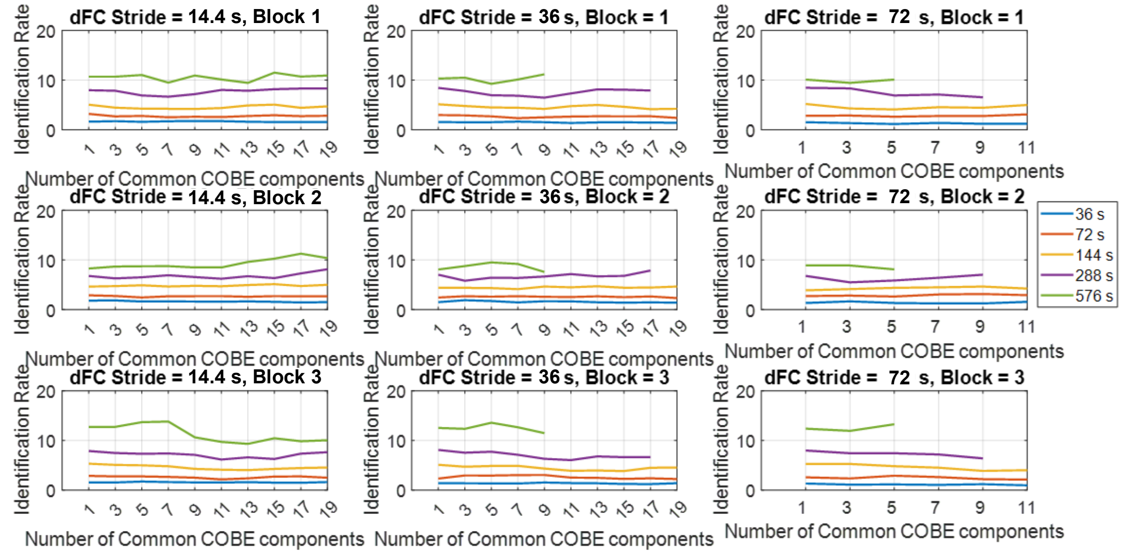

Figure S7: IR values computed for different values of window sizes, strides, and the number of common COBE components considering the Limbic network using Schaefer-HCP atlas.

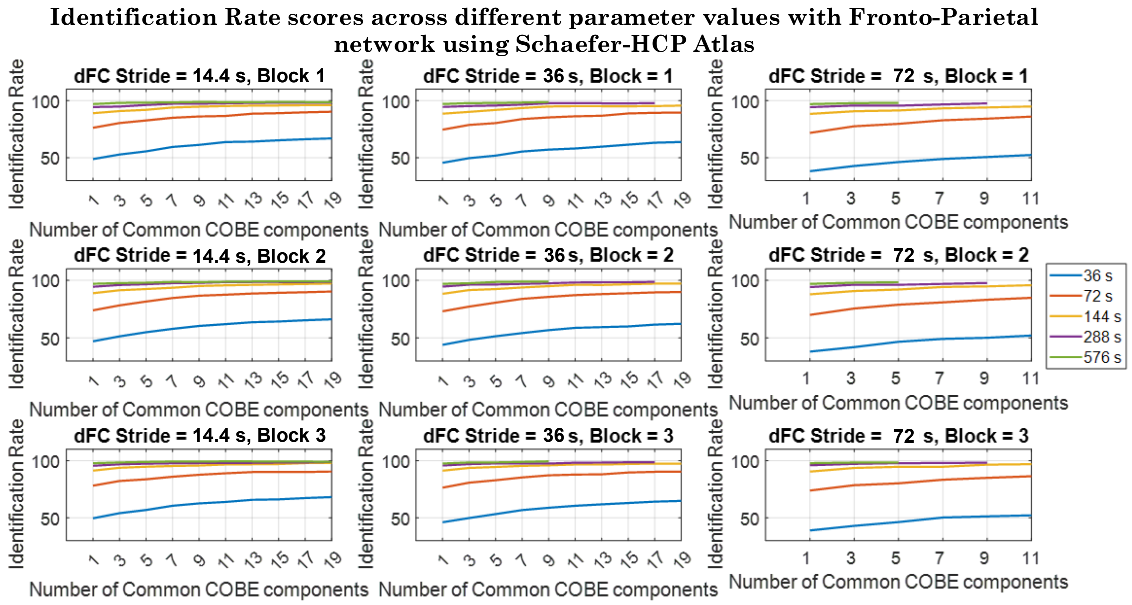

Figure S8: IR values computed for different values of window sizes, strides, and the number of common COBE components considering the Fronto-Parietal network using Schaefer-HCP atlas.

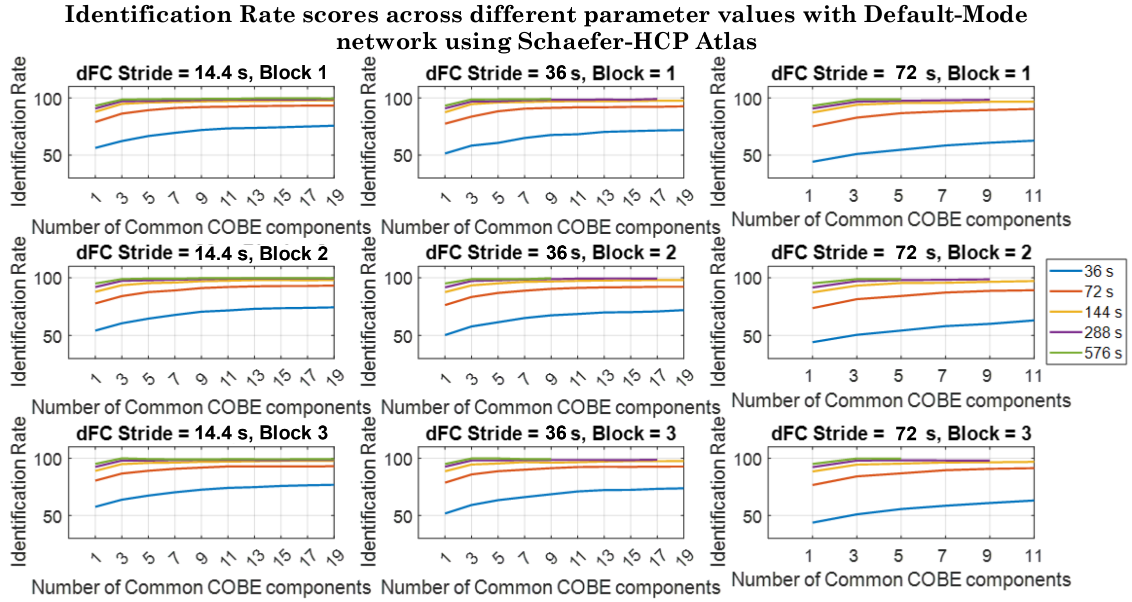

Figure S9: IR values computed for different values of window sizes, strides, and the number of common COBE components considering the Default-Mode network using Schaefer-HCP atlas.

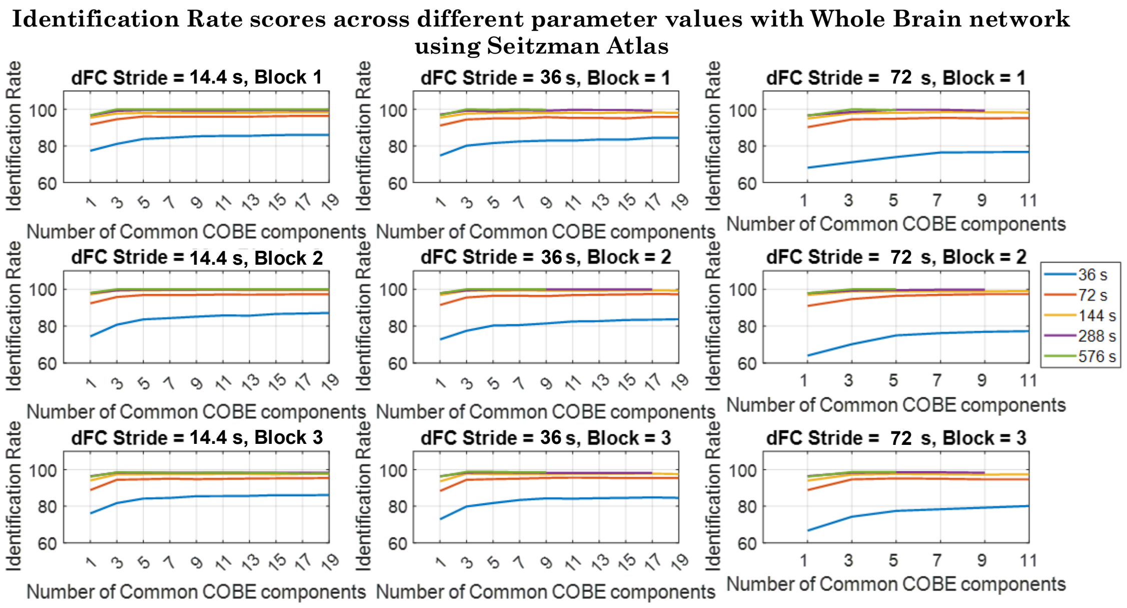

Figure S10: IR values computed for different values of window sizes, strides, and the number of common COBE components considering the Whole Brain network using Seitzman atlas.

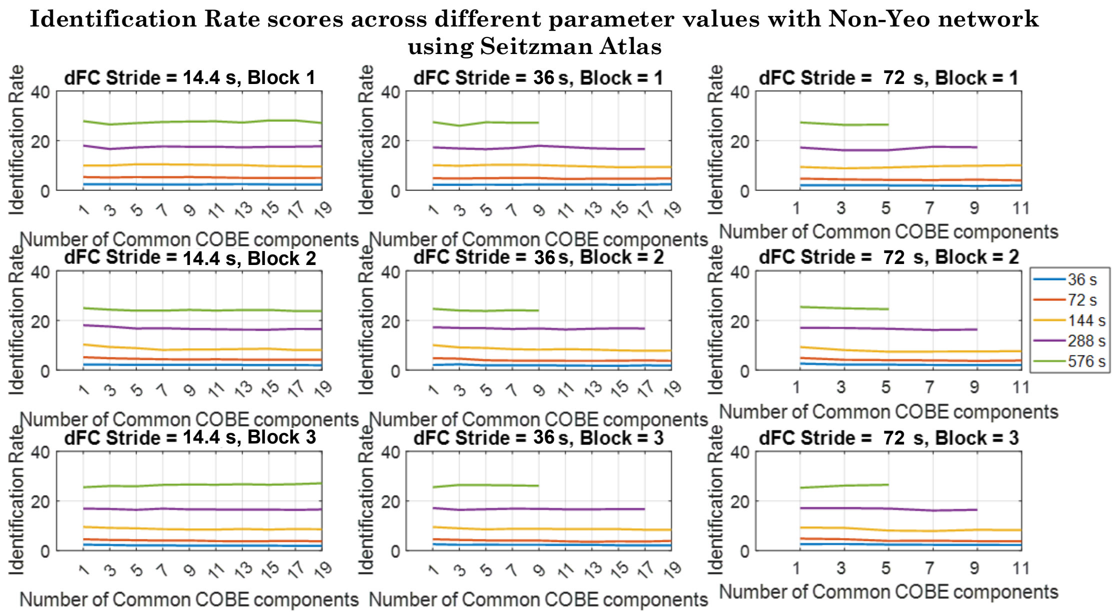

Figure S11: IR values computed for different values of window sizes, strides, and the number of common COBE components considering the Non-Yeo network using Seitzman atlas.

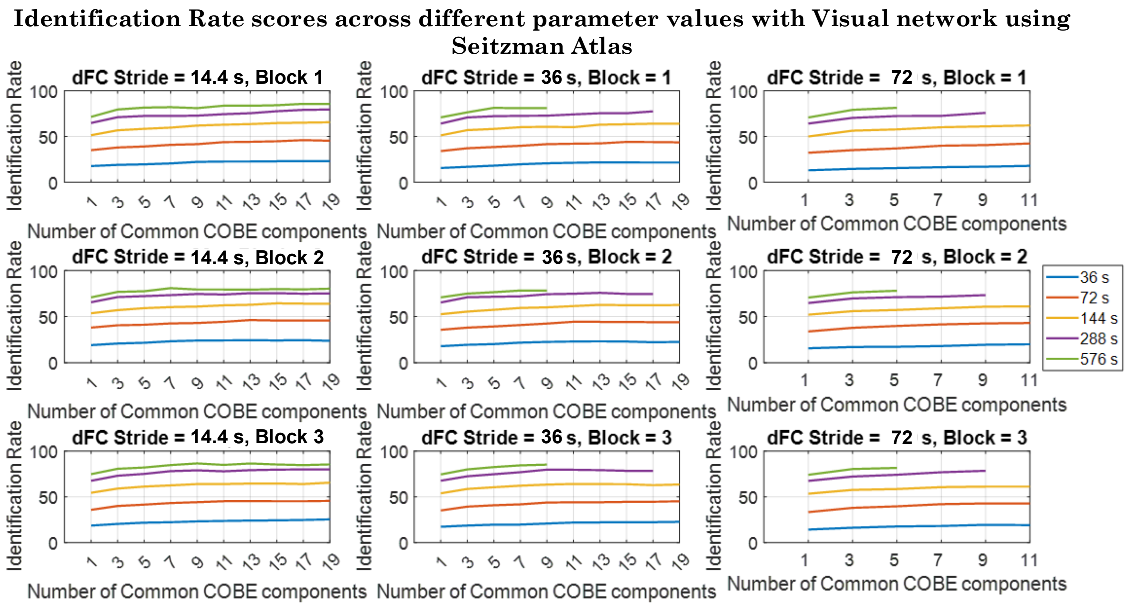

Figure S12: IR values computed for different values of window sizes, strides, and the number of common COBE components considering the Visual network using Seitzman atlas.

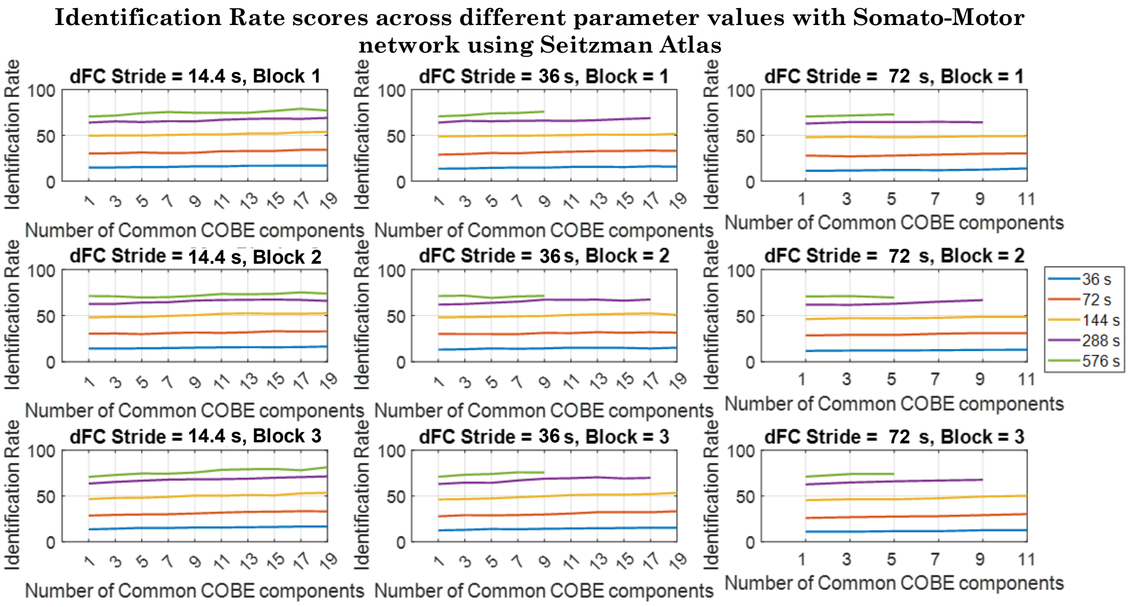

Figure S13: IR values computed for different values of window sizes, strides, and the number of common COBE components considering the Somato Motor network using Seitzman atlas.

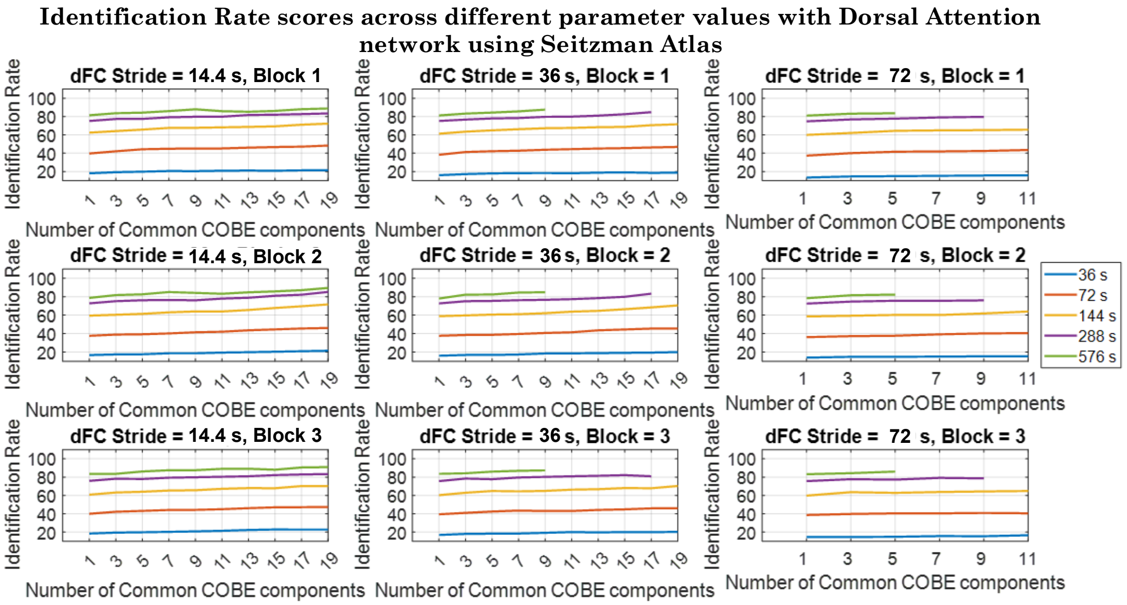

Figure S14: IR values computed for different values of window sizes, strides, and the number of common COBE components considering the Dorsal Attention network using Seitzman atlas.

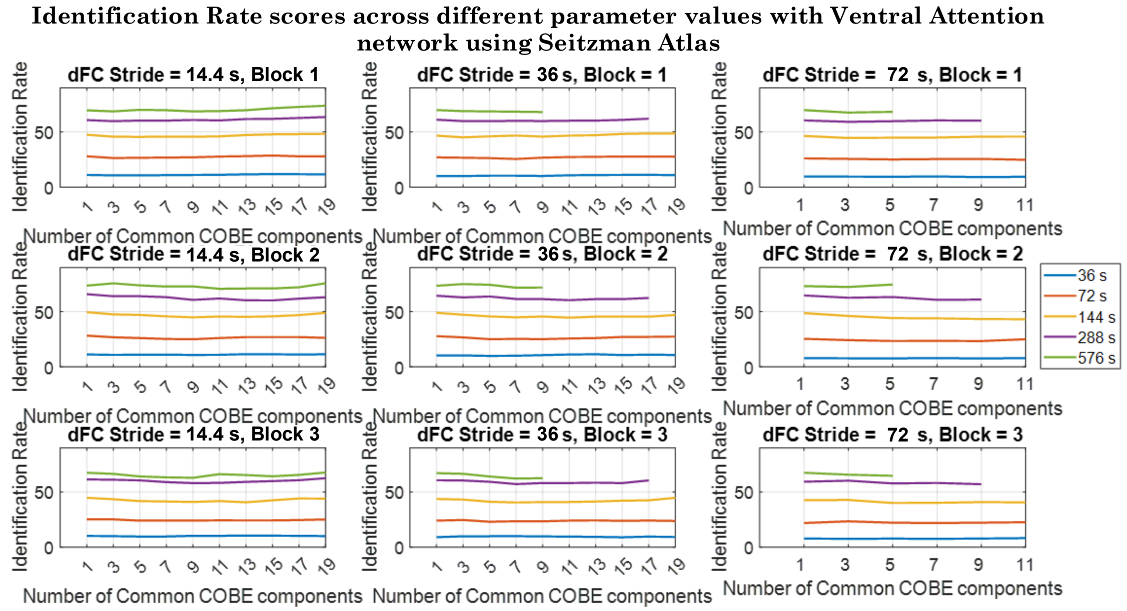

Figure S15: IR values computed for different values of window sizes, strides, and the number of common COBE components considering the Ventral Attention network using Seitzman atlas.

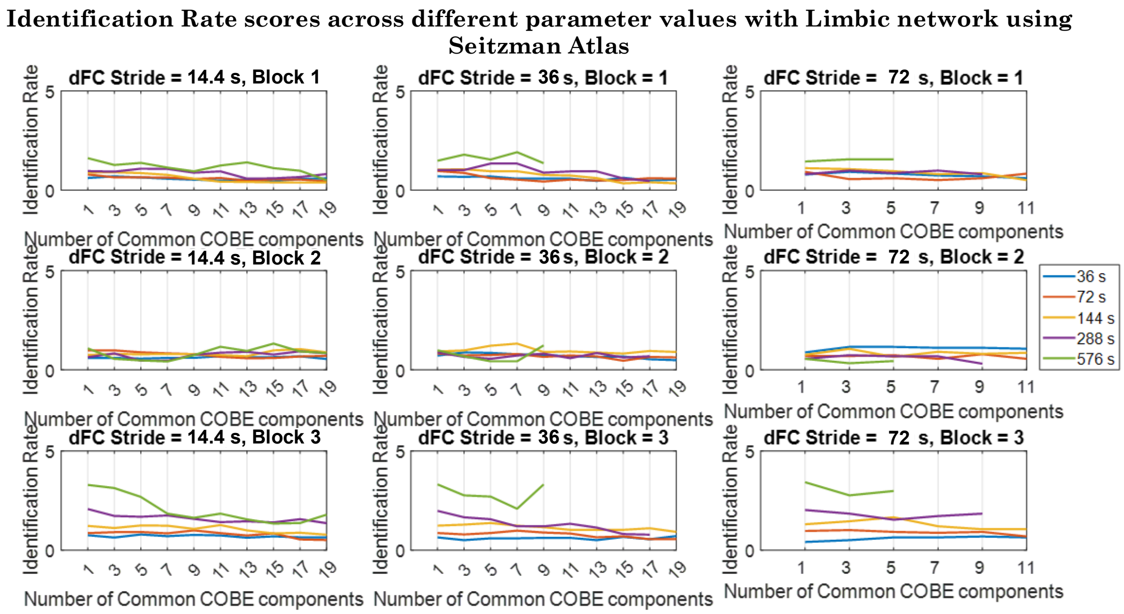

Figure S16: IR values computed for different values of window sizes, strides, and the number of common COBE components considering the Limbic network using Seitzman atlas.

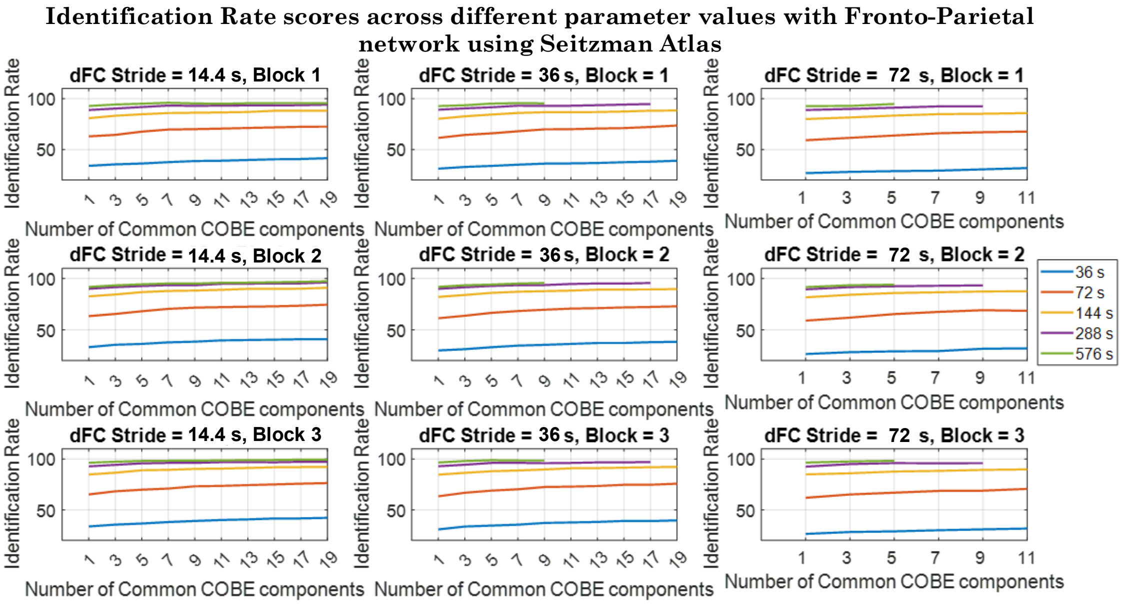

Figure S17: IR values computed for different values of window sizes, strides, and the number of common COBE components considering the Fronto-Parietal network using Seitzman atlas.

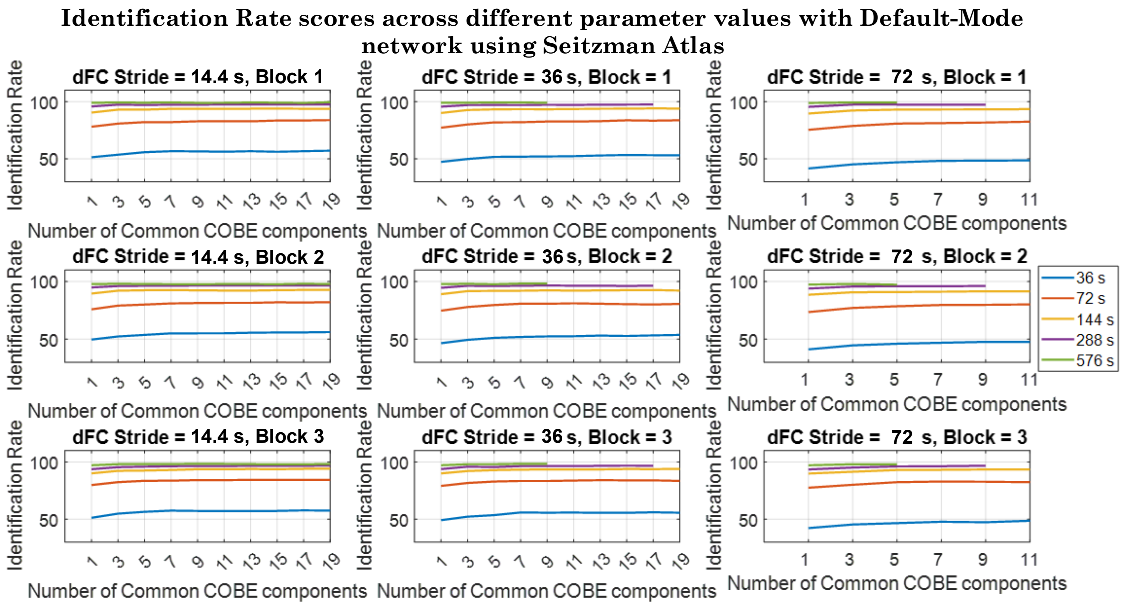

Figure S18: IR values computed for different values of window sizes, strides, and the number of common COBE components considering the Default Mode network using Seitzman atlas.

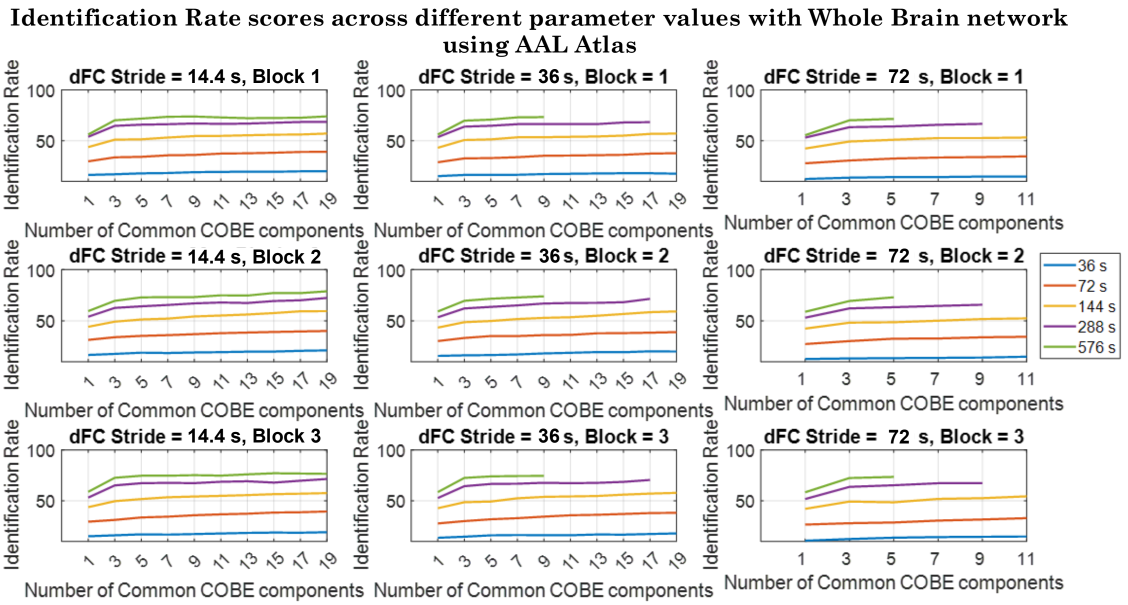

Figure S19: IR values computed for different values of window sizes, strides, and the number of common COBE components considering the Whole Brain network using AAL atlas.

Identification Rate scores across different parameter values with Non-Yeo network using AAL Atlas

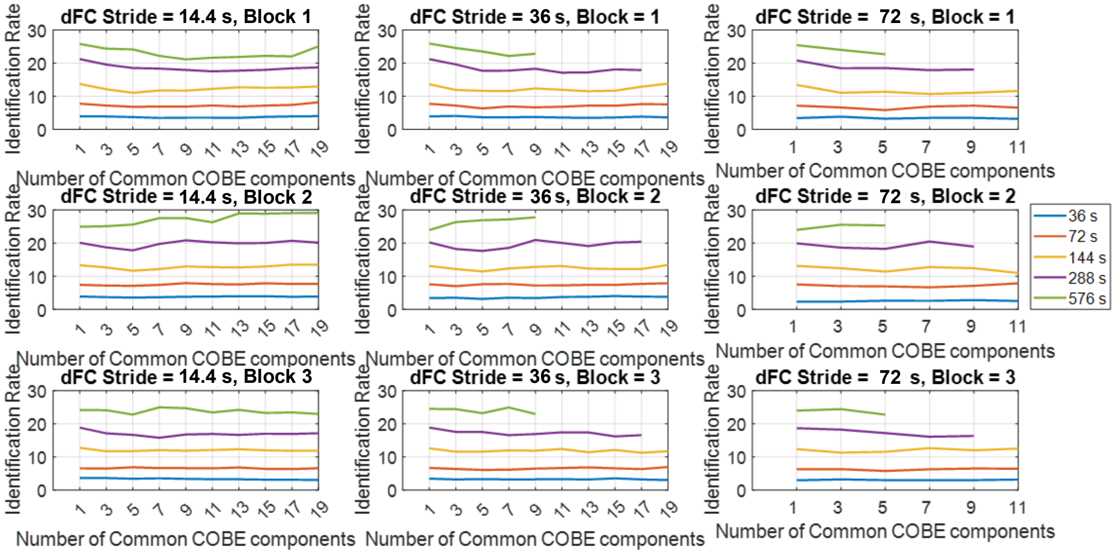

Figure S20: IR values computed for different values of window sizes, strides, and the number of common COBE components considering the Non-Yeo network using AAL atlas.

Identification Rate scores across different parameter values with Visual network using AAL Atlas

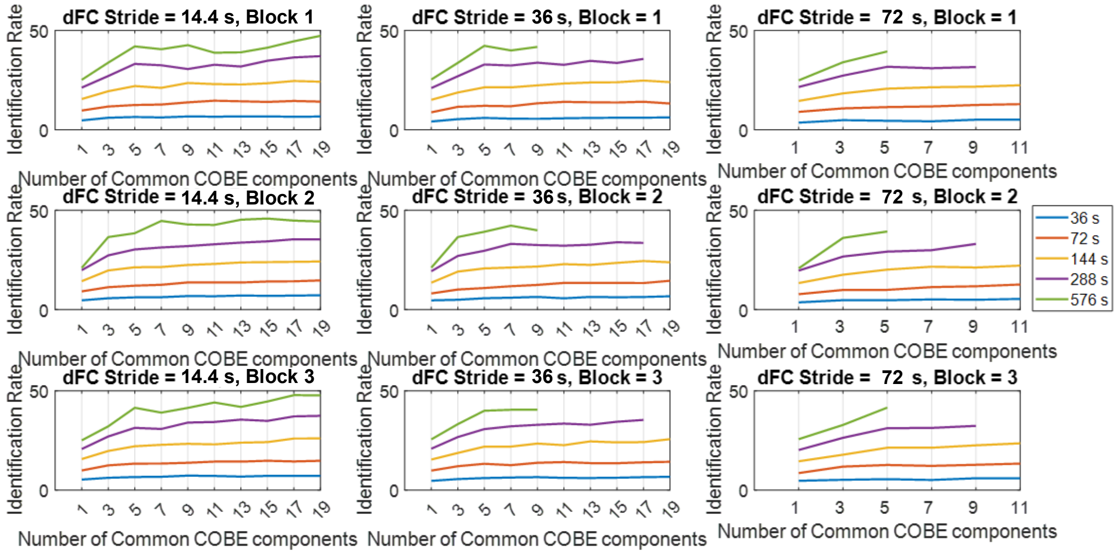

Figure S21: IR values computed for different values of window sizes, strides, and the number of common COBE components considering the Visual network using AAL atlas.

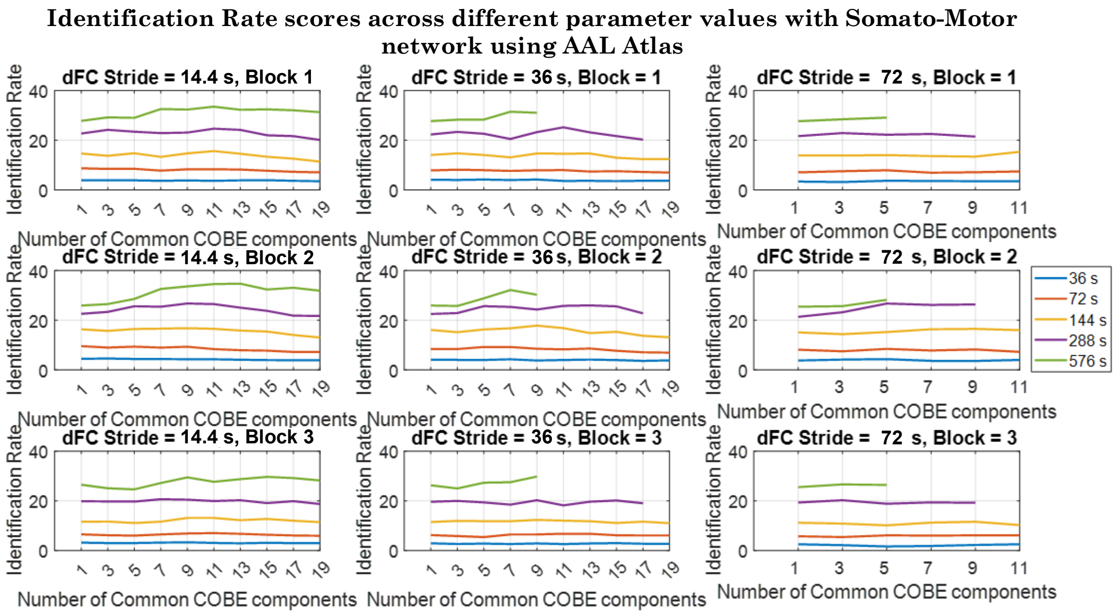

Figure S22: IR values computed for different values of window sizes, strides, and the number of common COBE components considering the Somato-Motor network using AAL atlas.

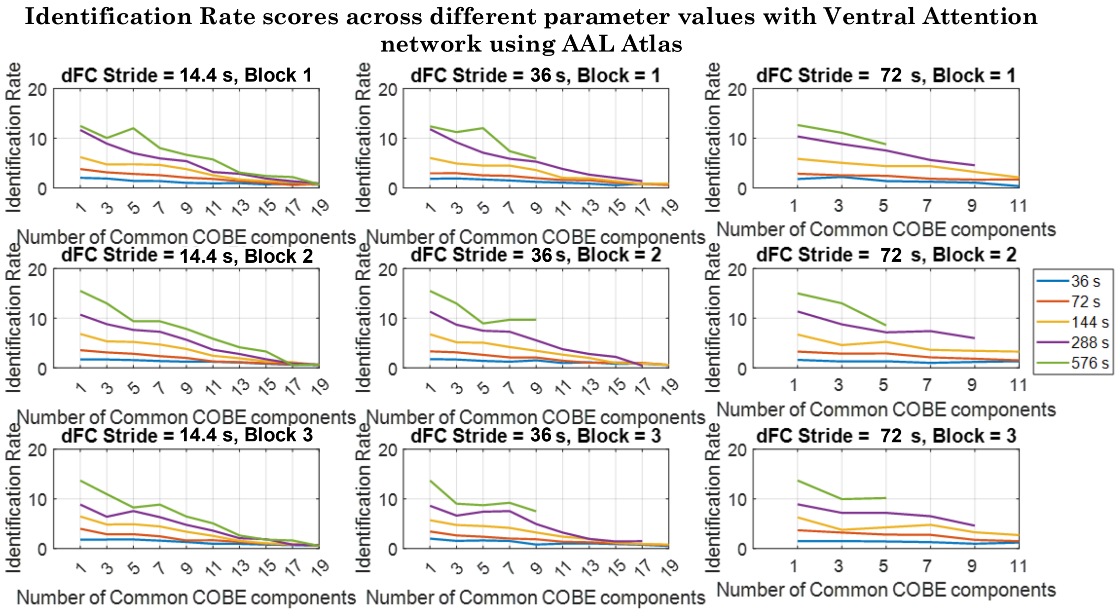

Figure S23: IR values computed for different values of window sizes, strides, and the number of common COBE components considering the Ventral Attention network using AAL atlas.

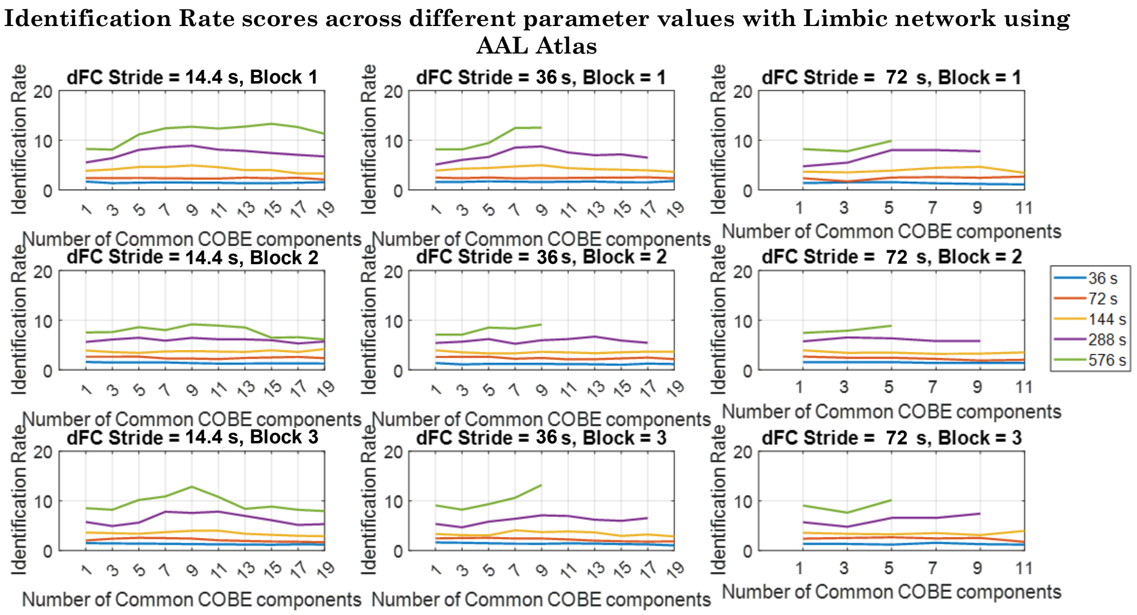

Figure S24: IR values computed for different values of window sizes, strides, and the number of common COBE components considering the Limbic network using AAL atlas.

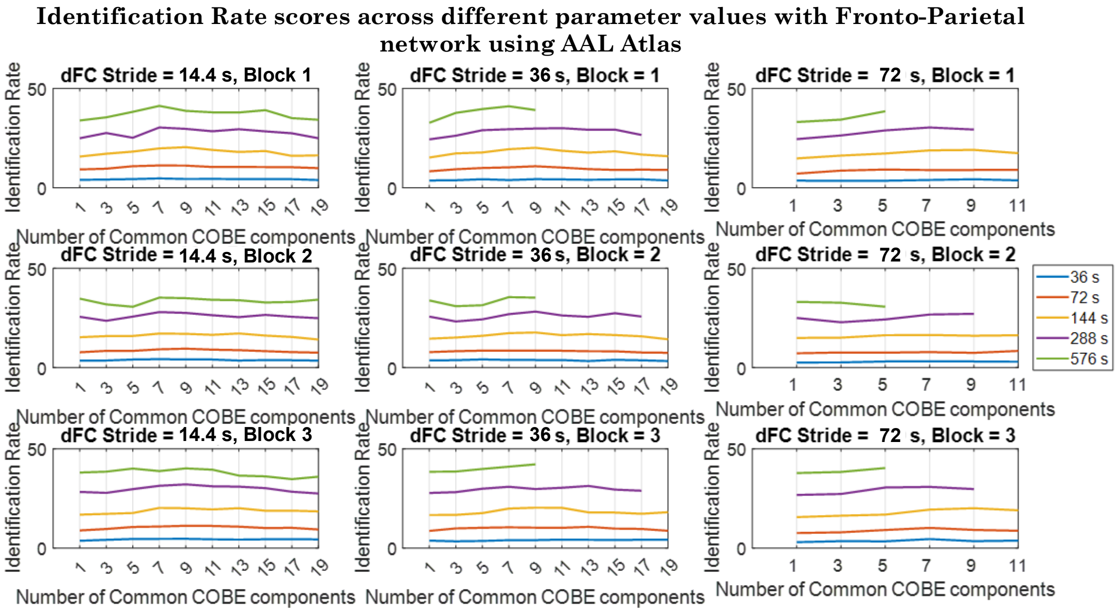

Figure S25: IR values computed for different values of window sizes, strides, and the number of common COBE components considering the Fronto-Parietal network using AAL atlas.

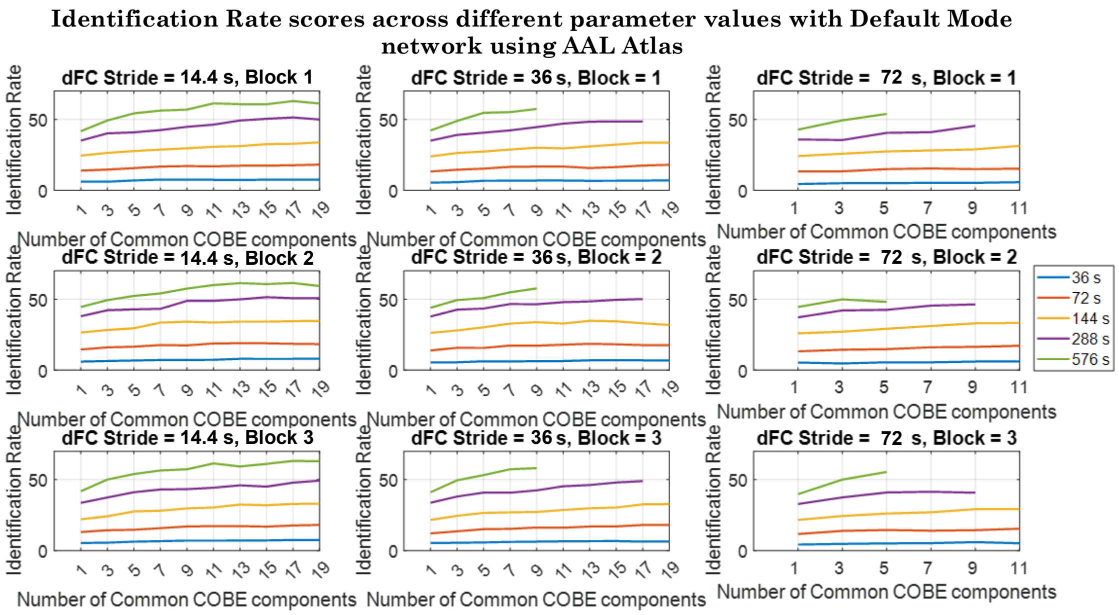

Figure S26: IR values computed for different values of window sizes, strides, and the number of common COBE components considering the Default Mode network using AAL atlas.

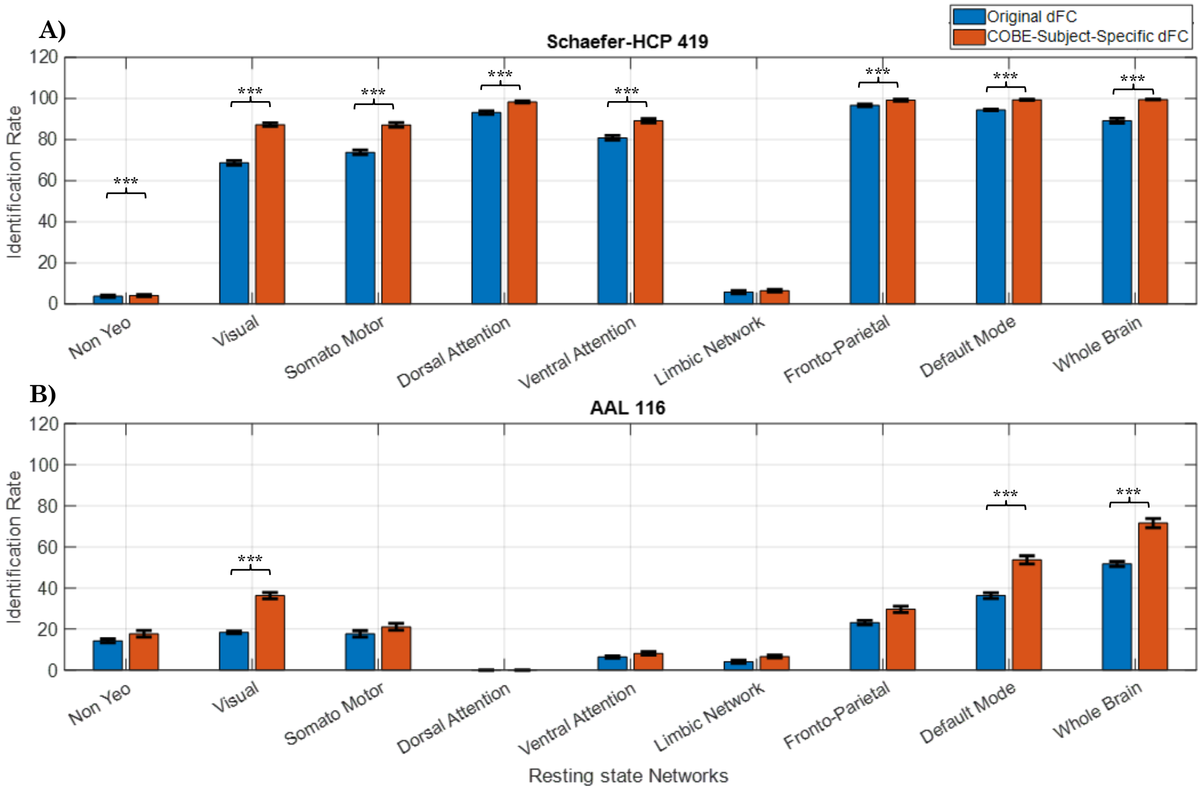

Figure S27 : IR scores comparison between A) Schaefer-HCP atlas and B) AAL atlas. The parameters that maximized the IR scores were chosen. T-values and corresponding p-values were computed using the 5x2cv method. Error bars represent mean $\pm$ std across the 10 permutations. *** → $p < \alpha_{corrected}$ ($\alpha_{corrected} = \frac{0.05}{9} = 0.00556$)

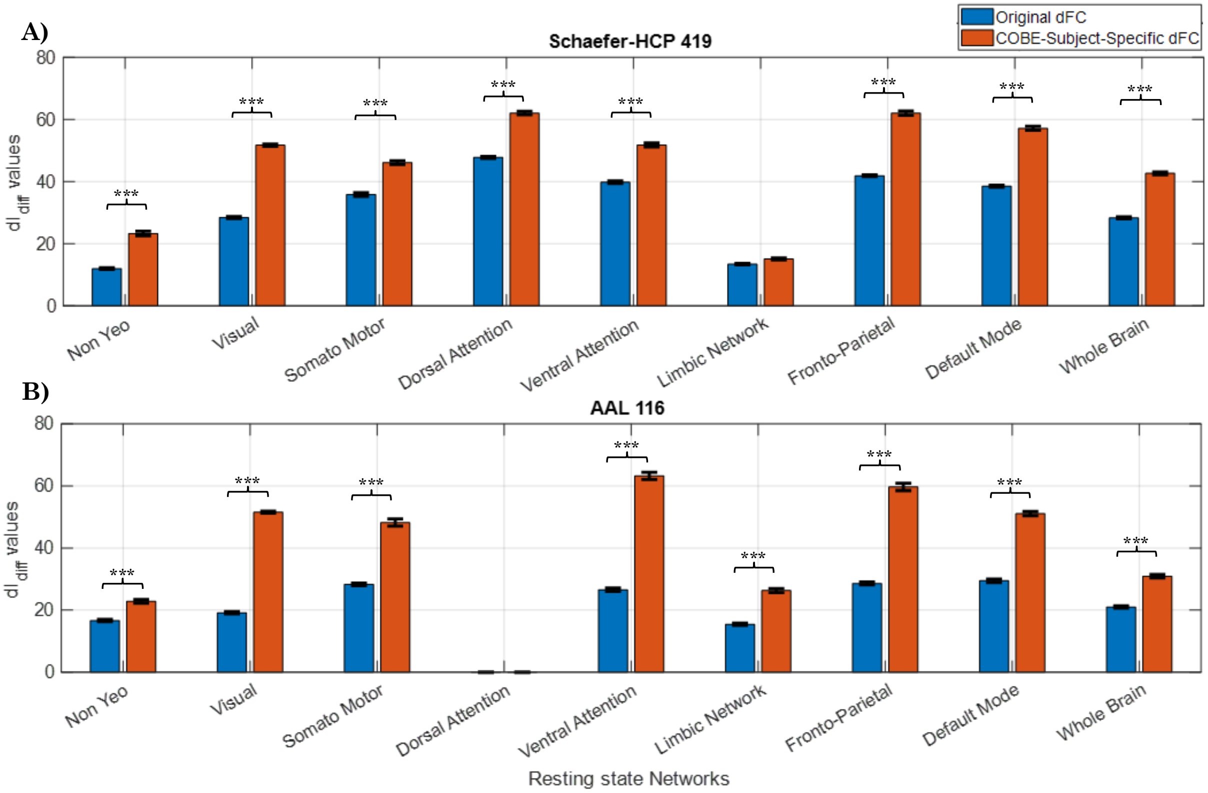

Figure S28 : dI_{diff} scores comparison between A) Schaefer-HCP atlas and B) AAL atlas.

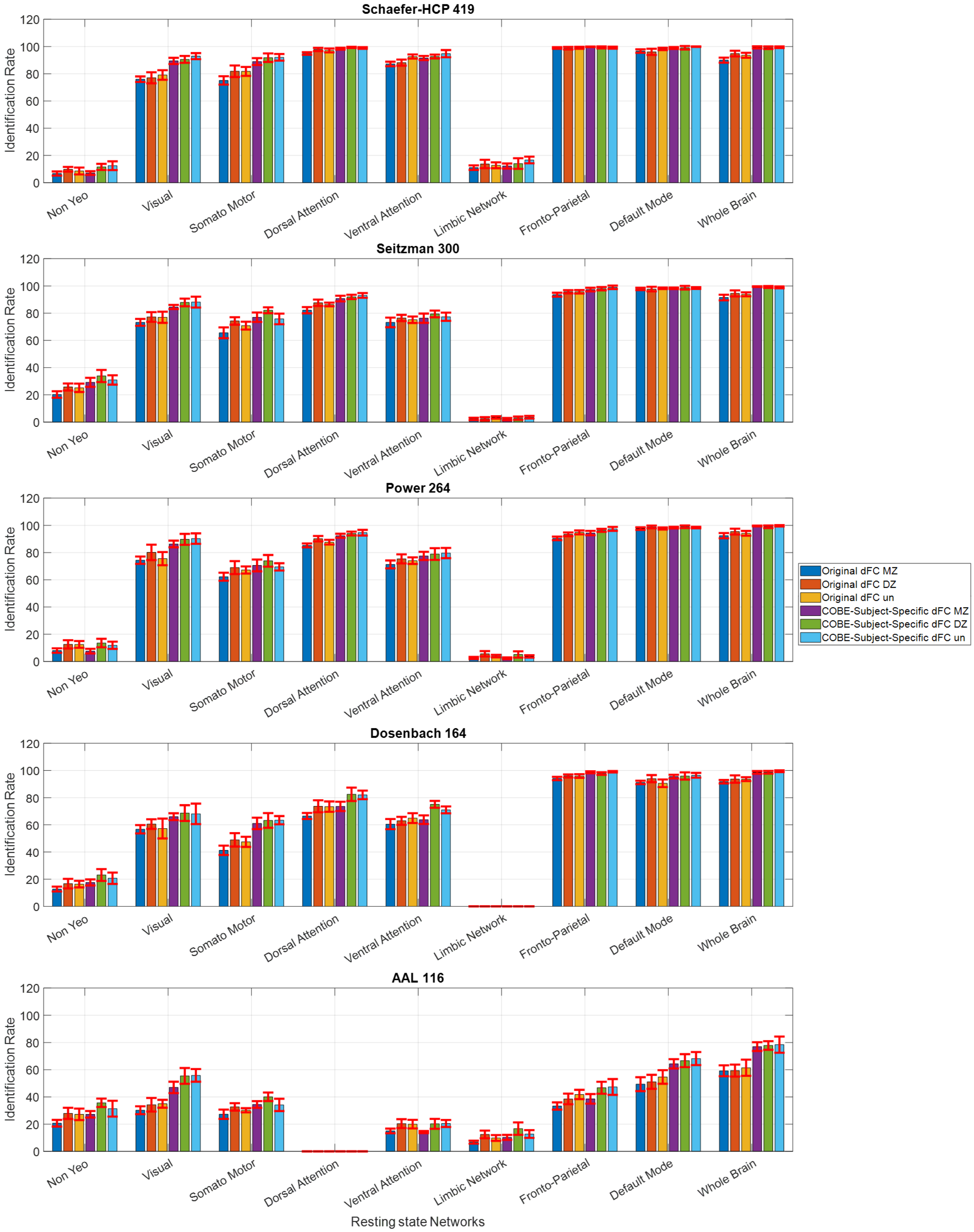

Figure S29: 5x2 Cross validation IR scores across the different atlases for Monozygotic (MZ), Dizygotic (DZ) and unrelated subject groups (un).

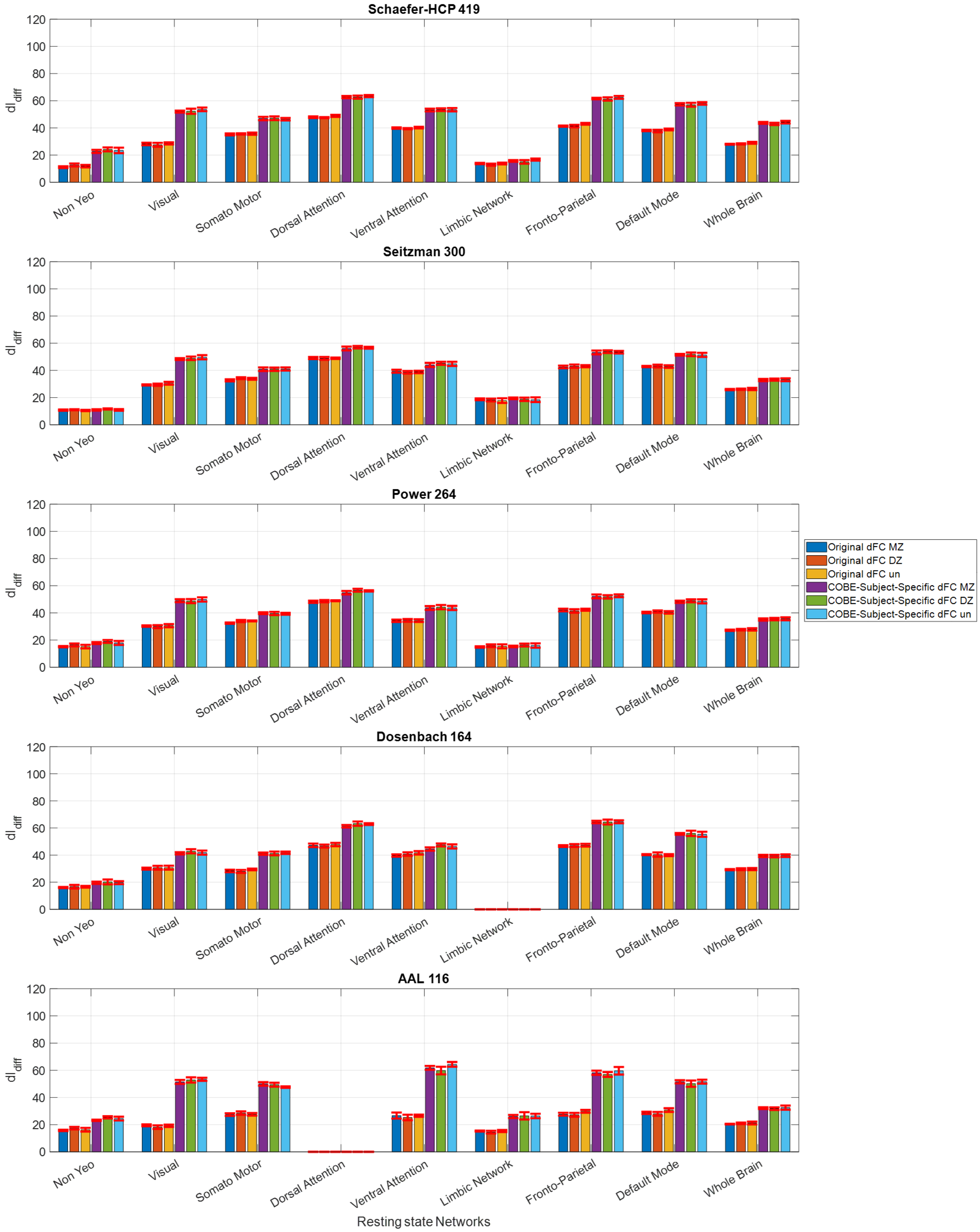

Figure S30: 5x2 Cross validation dI_{diff} scores across the different atlases for Monozygotic (MZ), Dizygotic (DZ) and unrelated subject groups (un).
